# Dynamic relationship between cerebrospinal fluid immune cells and tissue damage markers in multiple sclerosis

**DOI:** 10.1093/braincomms/fcaf387

**Published:** 2025-11-05

**Authors:** Sina Zaic, Theresa König, Markus Ponleitner, Florian Gföllner, Sara Silvaieh, Nik Krajnc, Tandis Parvizi, Stefan Macher, Barbara Kornek, Paulus Rommer, Fritz Leutmezer, Gabriel Bsteh, Elisabeth Stögmann, Thomas Berger, Tobias Zrzavy

**Affiliations:** Department of Neurology, Medical University of Vienna, Vienna 1090, Austria; Comprehensive Center for Clinical Neurosciences and Mental Health, Medical University of Vienna, Vienna 1090, Austria; Department of Neurology, Medical University of Vienna, Vienna 1090, Austria; Comprehensive Center for Clinical Neurosciences and Mental Health, Medical University of Vienna, Vienna 1090, Austria; Department of Neurology, Medical University of Vienna, Vienna 1090, Austria; Comprehensive Center for Clinical Neurosciences and Mental Health, Medical University of Vienna, Vienna 1090, Austria; Department of Neurology, Medical University of Vienna, Vienna 1090, Austria; Comprehensive Center for Clinical Neurosciences and Mental Health, Medical University of Vienna, Vienna 1090, Austria; Department of Neurology, Medical University of Vienna, Vienna 1090, Austria; Comprehensive Center for Clinical Neurosciences and Mental Health, Medical University of Vienna, Vienna 1090, Austria; Department of Neurology, Medical University of Vienna, Vienna 1090, Austria; Comprehensive Center for Clinical Neurosciences and Mental Health, Medical University of Vienna, Vienna 1090, Austria; Department of Neurology, Medical University of Vienna, Vienna 1090, Austria; Comprehensive Center for Clinical Neurosciences and Mental Health, Medical University of Vienna, Vienna 1090, Austria; Department of Neurology, Medical University of Vienna, Vienna 1090, Austria; Comprehensive Center for Clinical Neurosciences and Mental Health, Medical University of Vienna, Vienna 1090, Austria; Department of Neurology, Medical University of Vienna, Vienna 1090, Austria; Comprehensive Center for Clinical Neurosciences and Mental Health, Medical University of Vienna, Vienna 1090, Austria; Department of Neurology, Medical University of Vienna, Vienna 1090, Austria; Comprehensive Center for Clinical Neurosciences and Mental Health, Medical University of Vienna, Vienna 1090, Austria; Department of Neurology, Medical University of Vienna, Vienna 1090, Austria; Comprehensive Center for Clinical Neurosciences and Mental Health, Medical University of Vienna, Vienna 1090, Austria; Department of Neurology, Medical University of Vienna, Vienna 1090, Austria; Comprehensive Center for Clinical Neurosciences and Mental Health, Medical University of Vienna, Vienna 1090, Austria; Department of Neurology, Medical University of Vienna, Vienna 1090, Austria; Comprehensive Center for Clinical Neurosciences and Mental Health, Medical University of Vienna, Vienna 1090, Austria; Department of Neurology, Medical University of Vienna, Vienna 1090, Austria; Comprehensive Center for Clinical Neurosciences and Mental Health, Medical University of Vienna, Vienna 1090, Austria; Department of Neurology, Medical University of Vienna, Vienna 1090, Austria; Comprehensive Center for Clinical Neurosciences and Mental Health, Medical University of Vienna, Vienna 1090, Austria

**Keywords:** multiple sclerosis, CSF, NFL, GFAP, immunophenotyping

## Abstract

Multiple sclerosis (MS) is characterized by immune-mediated demyelination and neurodegeneration. While cerebrospinal fluid (CSF) biomarkers can track tissue damage, the relationship between immune cell populations and tissue damage markers remains poorly understood. We performed comprehensive immunophenotyping of CSF samples from 63 participants [29 relapsing-remitting multiple sclerosis (RRMS), 7 primary progressive multiple sclerosis (PPMS), 27 patients with other suspected neurological diseases (OND)]. CSF levels of neurofilament light chain (NfL) and glial fibrillary acidic protein (GFAP) were measured using single-molecule array technology. Relationships between immune cell populations and biomarkers were assessed using partial correlation and multiple regression analyses, adjusting for age and sex. RRMS patients exhibited expanded lymphocyte populations compared with OND, with elevated CD3+ T cells (+5062 cells/mL, *P* < 0.0001) and CD19+ B cells (+180 cells/mL, *P* < 0.0001). Patients with multiple sclerosis showed an age-related shift in monocyte subsets, marked by increased CD14+CD16+ cells [r_SP_ = 0.670, (95% CI: 0.44–0.81), *P* = 0.0029]. During active relapse, naive CD4+ T cells demonstrated the strongest association with NfL [cumulative geometric mean ratio = 2.892 (95% CI: 1.352–6.188), *P* < 0.0001], contrasting with non-relapse states [GMR = 0.689 (95% CI: 0.449–1.057), *P* = 0.101]. This study identifies distinct immunological signatures in multiple sclerosis and demonstrates disease activity-dependent associations between specific immune cell populations and tissue damage markers. The relationship between classical monocytes and GFAP in controls suggests a previously unrecognized role for myeloid cells in physiological CNS homeostasis.

## Teaser

Zaic *et al*. report that relapsing-remitting multiple sclerosis patients show elevated cerebrospinal fluid T and B lymphocytes compared with controls, with age-related monocyte subset shifts. Disease activity-dependent associations between immune cell populations and neurodegeneration markers suggest distinct immunological signatures contribute to tissue damage across different multiple sclerosis activity states.

## Graphical Abstract

**Graphical Abstract:**
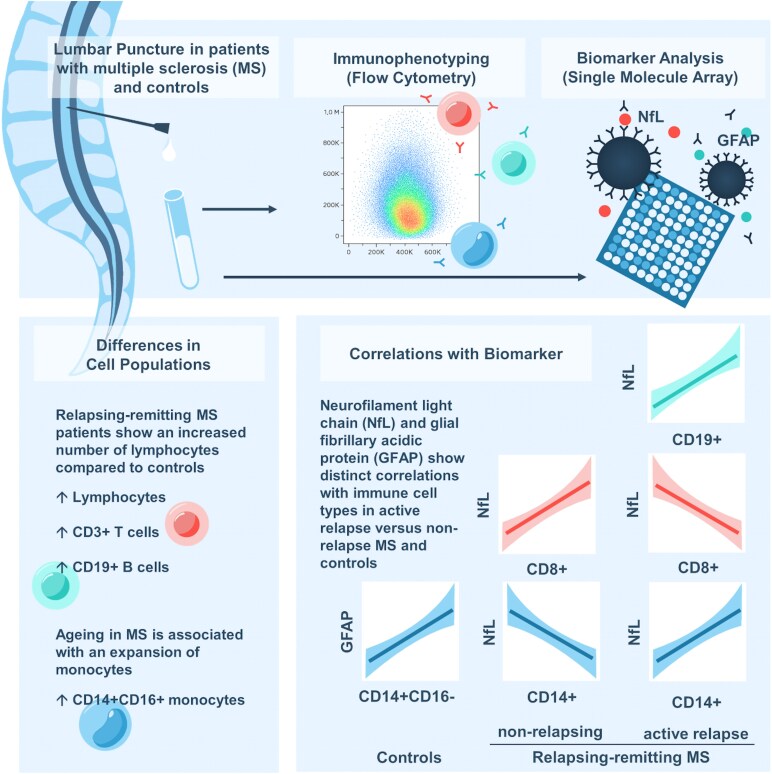

## Introduction

Multiple sclerosis (MS) is a complex immune-mediated disease characterized by chronic inflammation, demyelination, and neurodegeneration of the central nervous system (CNS). While traditionally classified into distinct clinical phenotypes (relapsing, secondary progressive, and primary progressive MS), emerging pathological evidence suggests that MS represents a disease continuum rather than discrete disease courses, with all pathological hallmarks present from the earliest stages, albeit in varying quantities.^1^ The pathogenesis of MS involves complex interactions between T and B lymphocytes, though their precise contributions remain incompletely understood.^2^ Acute inflammatory lesions are hypothesized to form when peripheral immune cells infiltrate the CNS, triggering an inflammatory cascade that leads to demyelination and, when occurring in eloquent areas, clinical relapses. In contrast, relapse-independent progression involves compartmentalized inflammation with lymphocyte accumulation in the meninges and Virchow-Robin spaces.^3,4^ This compartmentalized inflammation associates with multiple pathological processes, including enlargement of pre-existing lesions, cortical lesion formation, and diffuse neurodegeneration.^5^

The cerebrospinal fluid (CSF) constitutes a crucial interface in MS pathophysiology, functioning both as a dynamic trafficking corridor and reservoir for immune cells, while simultaneously providing a protective envelope around the CNS. Experimental evidence has demonstrated that CSF facilitates the initial entry of encephalitogenic immune cells during lesion formation, while the presence of oligoclonal bands and/or kappa free light chains, coupled with B cell antigen-driven affinity maturation and acquisition of tissue-resident cellular phenotypes, provides strong evidence for sustained compartmentalized intrathecal inflammation.^6-9^ Moreover, distinct molecular and cellular profiles have been identified in the CSF of MS patients, with subtle yet significant variations observed among different disease courses, suggesting disease-specific inflammatory signatures.^10,11^

Fluid biomarkers associated with disease activity and progression, particularly neurofilament light chain (NfL) and glial fibrillary acidic protein (GFAP) in CSF, have been demonstrated to reflect distinct pathological processes, with NfL capturing acute neuroaxonal injury and GFAP reflecting astrocytic activation, capturing some features of chronic smouldering compartmentalized inflammation.^12,13^ However, the relationship between these markers and specific immune cell populations in different MS phenotypes and disease states remains poorly understood.

These observations highlight the need for a comprehensive analysis of CSF immune cell populations and their relationship to disease progression markers across different MS phenotypes. The present study conducted a comprehensive immunophenotyping of CSF immune cell populations in MS patients, analysing multiple immune cell subsets while simultaneously measuring NfL and GFAP levels.

Our objectives were to: (i) identify immunological signatures of MS, (ii) characterize immune cell shifts during acute relapses, (iii) investigate age-related patterns in immune cell populations, and (iv) establish correlations between immune cell subsets and tissue damage markers.

A deeper understanding of these relationships may provide crucial insights into the heterogeneous mechanisms driving MS progression.

## Materials and methods

### Study cohort

Study participants were recruited from patients undergoing diagnostic lumbar punctures (LP) at the Department of Neurology, Medical University of Vienna. All participants provided informed consent. Multiple Sclerosis (MS) patients met the 2017 McDonald criteria and were treatment-naïve. Patients with other suspected neurological diseases (OND) had no systemic or CNS immunological/infectious/neurodegenerative diseases. Inclusion criteria for all participants included age ≥18 years and capacity to consent. Active relapse was defined as objectively confirmed symptoms of CNS inflammatory demyelination lasting ≥24 h, occurring within 2 weeks before LP, without fever or infection. Non-relapsing patients must not have had a relapse within the previous 3 months.

### CSF/WB processing

Fresh CSF samples were transported on ice to the laboratory within 15 min after LP and were screened using ChemStrip (20 μL); samples with ≥50 Erythrocytes/μL were excluded. CSF was centrifuged (400 g, 10 min, 4°C), and cells were counted after pellet resuspension. For staining, samples were incubated with Fc-Block (10 min) followed by the antibody cocktail (30 min) (Supplementary Table 1), washed, and centrifuged (400 g, 5 min, 4°C). The final pellet was resuspended in 200 μL stain buffer. WB samples underwent RBC lysis (1:10 dilution) before following the same processing steps as in CSF. In this study WB was solely used for gate setting without separate analysis. All samples were analysed using a Cytek Aurora flow cytometer (16UV-16V-14B-10YG-8R).

Spectral unmixing was performed using SpectroFlo® software (Version 3.1.0, Cytek Biosciences). The unmixed data were exported and analysed using FlowJo™ software (Version 10.9.0, BD Biosciences). Detailed gating strategies and cell population definitions are provided in Supplementary Fig. 1 and Table 1.^14^

### NfL/GFAP

CSF samples were obtained from our neurological biobank, where aliquots are stored at −70°C.^15^

CSF collected during cell staining preparation was frozen, later thawed, and analysed for NfL and GFAP using single-molecule array (SIMOA) Neurology 2 Plex B assay kits in a SIMOA SR-X Analyzer (Quanterix). All samples were processed simultaneously by a blinded investigator.

### Statistical analysis

Differences in demographic and clinical characteristics between groups were assessed using non-parametric tests, as indicated by the use of medians and interquartile ranges (IQR). Group comparisons for continuous variables were performed using Kruskal-Wallis tests, followed by appropriate post-hoc analyses. Fisher’s Exact Test was used for categorical variables, such as sex distribution across groups. For immune cell population analyses, between-group differences in both absolute counts and relative frequencies were evaluated. Age-related patterns were assessed using Spearman's rank correlation coefficients (r_SP_). Partial correlation analyses were employed using Spearman's rank correlation to examine relationships between immune cell populations and tissue damage markers (NfL and GFAP) while controlling for age. Multiple regression analyses were conducted to examine how immune cell populations related to biomarker levels (log-transformed NfL and GFAP), adjusting for age and sex. Log-log regression models were used where appropriate, adjusting for age and sex as covariates. For analyses involving relapse status, interaction terms were included to assess differential relationships between immune cell populations and biomarker levels during relapse and non-relapse states.

Model assumptions were systematically assessed: normality of residuals was evaluated using Q-Q plots and Shapiro-Wilk tests; homoscedasticity was assessed through residual-versus-fitted value plots; linearity was examined assessed through partial regression plots for quantitative predictors. NfL and GFAP values were log-transformed due to their right-skewed distributions, as verified by histograms and Shapiro-Wilk tests. Normality of the log-transformed data was confirmed.

For regression models with log-transformed outcomes (NfL and GFAP), beta coefficients were exponentiated and reported as geometric mean ratios with 95% confidence intervals. For continuous predictors that were log10-transformed, the geometric mean ratios represent the multiplicative change in the outcome associated with a 10-fold increase in the predictor.

Model performance was evaluated using adjusted *R*² values. All correlation analyses and regression analyses were corrected for multiple testing using the Benjamin-Hochberg correction. Statistical significance was set at *P* < 0.05 for all analyses, and only adjusted *P*-values are reported.

Sample size was determined by feasibility constraints of CSF collection and predefined inclusion criteria, aligning with the exploratory study design without a priori power calculations. Statistical analyses were performed using R (version 2024.9.0.375).

### Ethics

The study was approved by the ethics committee of the Medical University Vienna (ethical approval number: 1715/2023).

## Results

The study included 63 participants: 27 patients with other suspected neurological diseases (OND), 7 primary progressive multiple sclerosis (PPMS), and 29 relapsing-remitting multiple sclerosis (RRMS) patients (Table 1). Patients with PPMS were significantly older than both OND and RRMS groups. The cohort was predominantly female (59%), with similar sex distribution across groups. At sampling, 35% of patients with RRMS were experiencing an active relapse, and 14% had received high-dose methylprednisolone therapy before LP. Patients with RRMS showed significantly higher CSF cell counts compared with other groups (Table 1).

**Table 1:** Demographic and clinical characteristics stratified by diagnosis

Clinical and Demographic Characteristics						
Characteristic	*N*	Overall^a^	RRMS *N* = 29^a^	Co *N* = 27^a^	PPMS *N* = 7^a^	*P*-value^b^
Age (years)	63	35.0 (26.0, 47.0)	36.0 (27.0, 43.0)	31.0 (23.0, 45.0)	61.0 (52.0, 65.0)	0.006
Sex	63					0.76
f		37 (58.7%)	16 (55.2%)	16 (59.3%)	5 (71.4%)	
m		26 (41.3%)	13 (44.8%)	11 (40.7%)	2 (28.6%)	
CSF volume (mL)	63	11.5 (9.0, 14.0)	11.5 (9.5, 14.0)	11.5 (8.0, 13.5)	13.0 (8.5, 14.0)	0.80
Cell count (cells/µL)	63	3.0 (2.0, 8.0)	8.0 (4.0, 12.0)	2.0 (1.0, 3.0)	2.0 (2.0, 9.0)	<0.001
Oligoclonal bands	63					<0.001
Pos		34 (54.0%)	27 (93.1%)	0 (0.0%)	7 (100.0%)	
Neg		29 (46.0%)	2 (6.9%)	27 (100.0%)	0 (0.0%)	
EDSS score	36	1.3 (0.0, 3.0)	1.0 (0.0, 2.0)	NA	3.5 (3.0, 4.5)	
HDMP treatment	29					
No		25 (86.2%)	25 (86.2%)	NA	NA	
Yes		4 (13.8%)	4 (13.8%)	NA	NA	
Active relapse	29					
No		19 (65.5%)	19 (65.5%)	NA	NA	
Yes		10 (34.5%)	10 (34.5%)	NA	NA	

Cells/µL: Cells per microliter; Co: Control group; CSF: Cerebrospinal fluid; EDSS: Expanded Disability Status Scale; f: Female; HDMP: High-dose methylprednisolone; m: Male; *N*: Number of participants; NA: Not available; PPMS: Primary Progressive Multiple Sclerosis; RRMS: Relapsing-Remitting Multiple Sclerosis.

^a^Median (Q1, Q3); *n* (%).

^b^Kruskal-Wallis rank sum test.

**Figure 1:**
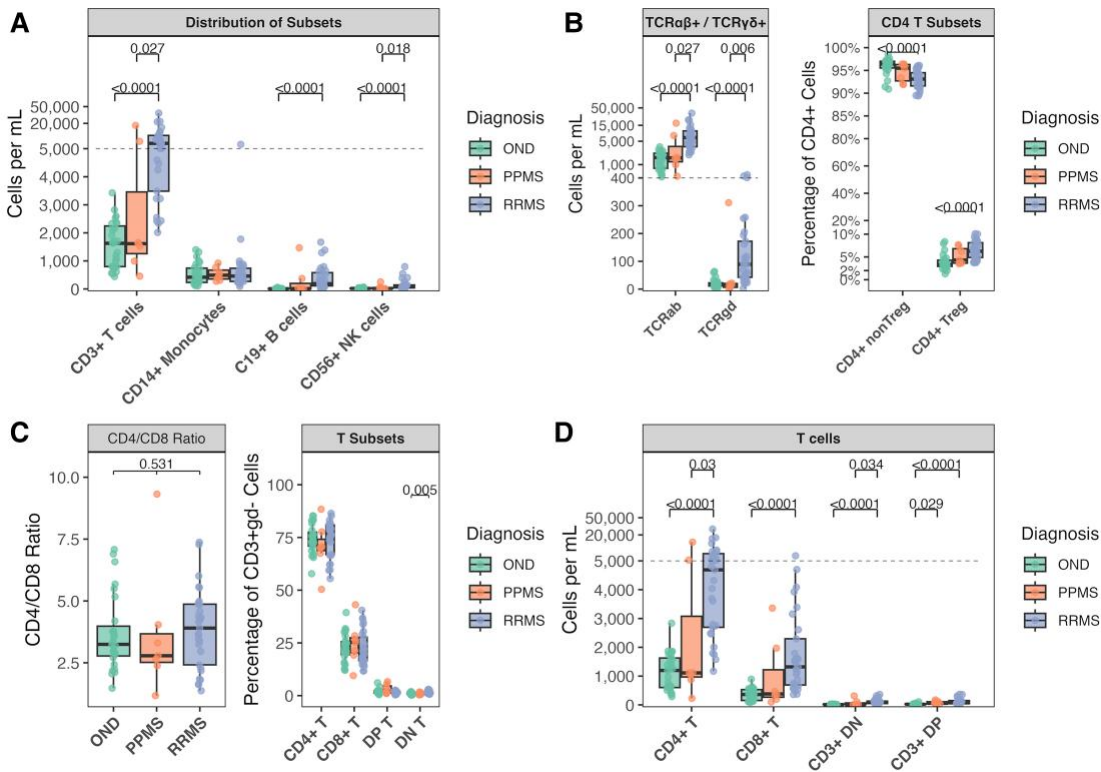
**Cerebrospinal fluid immune cell populations.** (**A**) Absolute counts of major immune lineages (CD3+ T cells, CD19+ B cells, CD14+ monocytes, CD56+ natural killer cells) in RRMS, PPMS, and other neurological diseases (OND). (**B**) T cell receptor alpha-beta positive (TCRαβ+) and T cell receptor gamma-delta positive (TCRγδ+) T cell distribution and frequencies of conventional CD4+ and regulatory T cells (Tregs). (**C**) CD4/CD8 ratios and relative proportions. (**D**) Absolute counts of CD4+ and CD8+ T cells. Statistical comparisons were performed using pairwise Wilcoxon rank-sum tests with Benjamini-Hochberg correction for multiple testing. Each data point represents an individual patient sample. Sample sizes: RRMS (*N* = 29), PPMS (*N* = 7), OND (*N* = 27). The boxes show the interquartile range with the median line, while the whiskers extend to the largest and smallest values within 1.5 × IQR from the box edges.

Analysis of immune cell populations revealed distinct alterations in the CSF of MS patients compared with OND, with notable differences between patients with RRMS and PPMS. Patients with RRMS exhibited significant expansion of CD3+ T cells compared with OND in both absolute count [+5062 cells/mL, (IQR: OND: 1448.50—RRMS: 6820.00), *P* < 0.0001] and frequency [+14.0%, (IQR: OND: 20.55—RRMS: 6.60), *P* < 0.0001], with similar elevations observed relative to patients with PPMS [absolute: +5074 cells/mL, (IQR: PPMS: 3305.00—RRMS: 6820.00), *P* = 0.027; frequency: +4.10%, (IQR: PPMS: 20.00—RRMS: 6.60), *P* = 0.042]. CD19+ B cells were significantly elevated in RRMS compared with OND [absolute: +180 cells/mL, (IQR: OND: 11.40—RRMS: 470.90), *P* < 0.0001; frequency: +2.64%, (IQR:OND: 0.39—RRMS:3.31), *P* < 0.0001]. While absolute CD14+ monocyte counts were stable across groups, their frequency was significantly reduced in patients with RRMS versus both OND [−17.1%, (IQR: OND:21.70—RRMS: 7.54), *P* < 0.0001] and PPMS [−9.33%, (IQR: PPMS: 22.06—RRMS: 7.54), *P* = 0.028]. CD56+ NK cells demonstrated significant increase in the RRMS cohort compared with both OND [+53.7 cells/mL, (IQR: OND: 17.70—RRMS: 102.80), *P* < 0.0001] and patients with PPMS [+54.3 cells/mL, (IQR: PPMS:31.94—RRMS: 102.80), *P* = 0.018] (Fig. 1A).

Within the T cell compartment, both TCRαβ+ and TCRγδ+ T cells showed significant expansion in RRMS compared with both OND [TCRαβ+: +4828 cells/mL, (IQR: OND: 1415.50—RRMS: 6764.00), *P* < 0.0001; TCRγδ+: +72.1 cells/mL, (IQR: OND: 11.40—RRMS: 129.10), *P* <0.0001] and patients with PPMS [TCRαβ+: +4858 cells/mL, (IQR: PPMS: 3296.50—RRMS: 6764.00), *P* = 0.027; TCRγδ+: +72.2 cells/mL, (IQR:PPMS: 13.20—RRMS: 129.10), *P* = 0.006] (Fig. 1B).

CD4+ T cells showed substantial increases in RRMS compared with OND, with both conventional T cells [+3136 cells/mL, (IQR: OND: 999.00—RRMS: 4529.00), *P* < 0.0001] and regulatory T cells (Tregs) being increased [+241 cells/mL, (IQR: OND: 26.25—RRMS: 288.00), *P* <0.0001]. Notably, Tregs showed also a proportional increase [+2.92%, (IQR: OND:1.4—RRMS:3.2), *P* < 0.0001] (Fig. 1B–D).

**Figure 2:**
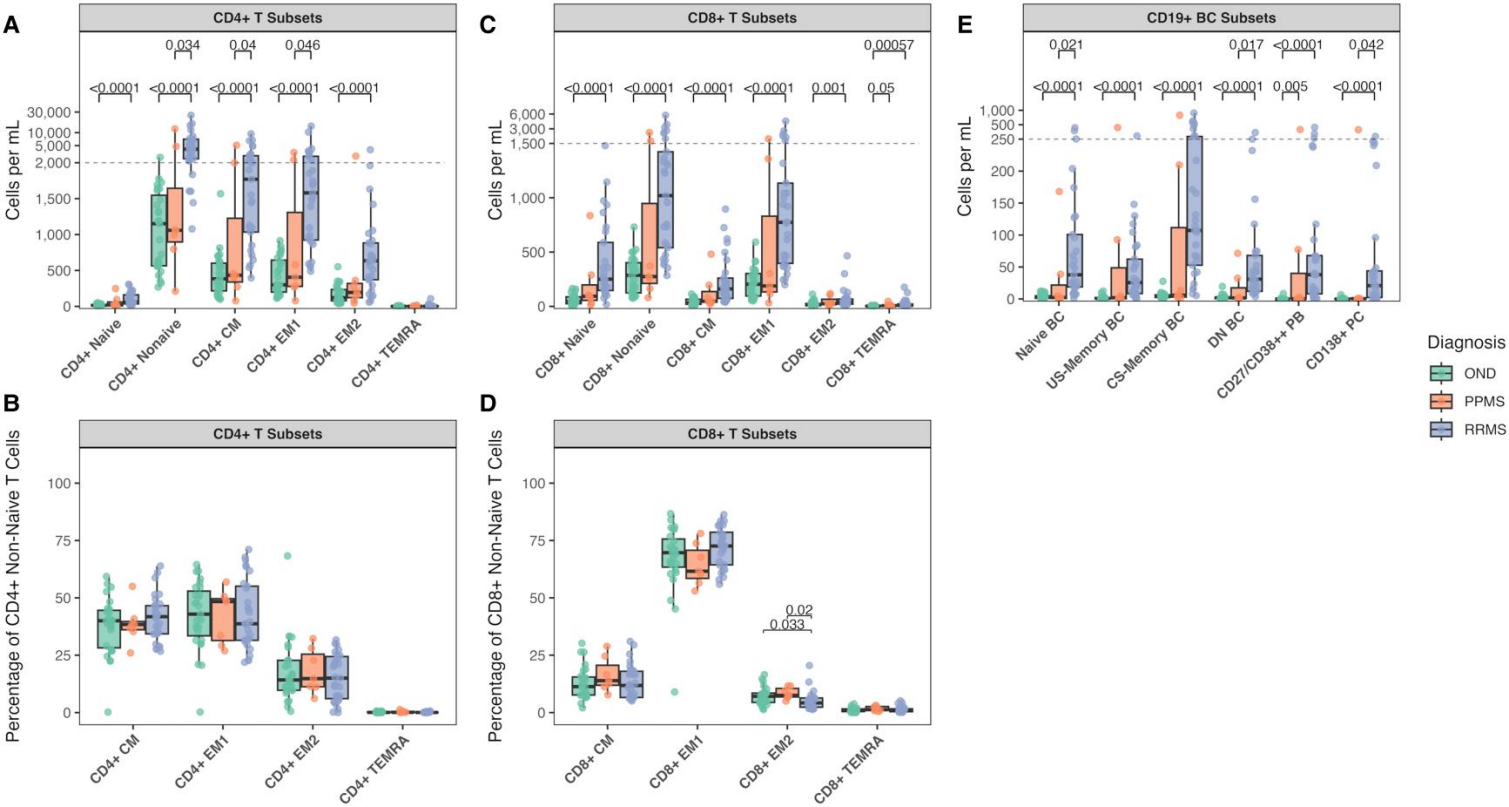
**Expansion of T and B lymphocyte populations in MS.** Panels are arranged **A**, **C**, **E** (top row) and **B**, **D** (bottom row). (**A**) Absolute counts of CD4+ T cell subsets showing significant expansion of naive, central memory, effector memory 1(EM1), and effector memory 2 (EM2) populations in RRMS versus other neurological diseases (OND) and PPMS. (**B**) Frequencies of memory CD4+ T cell subsets demonstrating preserved proportional distribution despite absolute changes. (**C**) Absolute counts of CD8+ T cell populations revealing expansion of both naive and memory subsets in RRMS. (**D**) Relative distributions of CD8+ memory T cell subsets showing maintained proportions. (**E**) Absolute counts of B cell subsets demonstrating significant expansion of naive, class-switched memory, and plasma cells in RRMS. Statistical comparisons were performed using pairwise Wilcoxon rank-sum tests with Benjamini-Hochberg correction for multiple testing. Each data point represents an individual patient sample. Sample sizes: RRMS (*N* = 29), PPMS (*N* = 7), OND (*N* = 27). The boxes show the interquartile range with the median line, while the whiskers extend to the largest and smallest values within 1.5 × IQR from the box edges.

CD4+ subsets in RRMS showed comprehensive expansion compared with OND, including naive [+45.4 cells/mL, (IQR: OND:18.45—RRMS: 125.10), *P* < 0.0001], central memory (CM) [+1386 cells/mL, (IQR: OND: 385.00—RRMS:1872.00), *P* < 0.0001], effector memory 1 (EM1) [+1280 cells/mL, (IQR: OND: 445.00—RRMS:1896.00), *P* < 0.0001], and effector memory 2 (EM2) [+514 cells/mL, (IQR: OND: 156.35—RRMS: 511.70), *P* < 0.0001] T cells (Fig. 2A and B).

Notably, differences between RRMS and PPMS were significant for CM [+1336 cells/mL, (IQR: PPMS: 888.00—RRMS: 1872.00), *P* = 0.040] and EM 1 [+1174 cells/mL, (IQR:PPMS: 1167.50—RRMS: 1896.00), *P* = 0.046] populations.

CD8+ T cells demonstrated significant expansion in RRMS versus OND [+960 cells/mL, (IQR: OND:377.00—RRMS: 1605.00), *P* < 0.0001], affecting both naive [+207 cells/mL, (IQR:OND: 62.10—RRMS: 441.80), *P* <0.0001] and memory populations [+734 cells/mL, (IQR:OND: 277.15—RRMS: 882.84), *P* < 0.0001] (Fig. 2C and D).

In the B cell compartment, all major subsets showed significant expansion in RRMS versus OND, including naive B cells [+35.3 cells/mL, (IQR:OND: 3.80—RRMS: 82.00), *P* < 0.0001], class-switched memory B cells [+104 cells/mL, (IQR: OND: 3.77—RRMS: 231.60), *P* < 0.0001], and plasma cells [+20.9 cells/mL, (IQR: OND: 0.33—RRMS: 41.46), *P* < 0.0001] (Fig. 2E). Plasmablasts showed consistent elevation in both RRMS [absolute: +38.0 cells/mL, (IQR: OND: 0—RRMS: 59.80), *P* < 0.0001] and PPMS [absolute: +0.22 cells/mL, (IQR: OND: 0—PPMS: 39.94] compared with OND].

**Figure 3:**
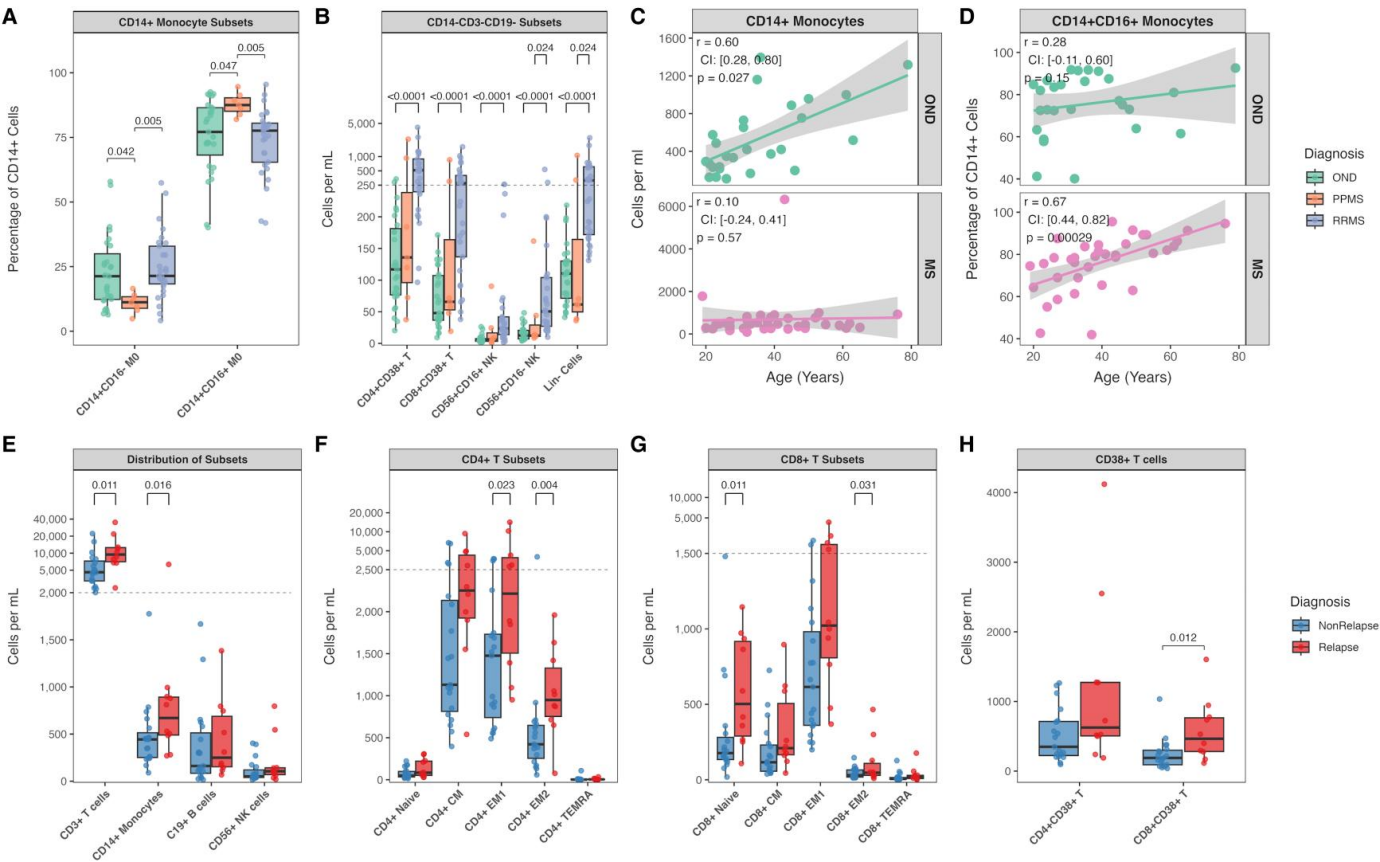
**Immune cell dynamics across MS, relapses, and age.** (**A**) Monocyte subset frequencies showing decreased (CD14+CD16−) monocytes in PPMS versus other neurological diseases (OND) and increased non-classical (CD14+CD16+) monocytes in PPMS versus RRMS. (**B**) Natural killer (NK) cell subsets and lineage-negative cells (CD3−CD14−CD19−) demonstrating expansion in RRMS versus OND, including both CD56+CD16+ and CD56+CD16− NK populations (**C**) Positive correlation between absolute CD14+ monocyte counts and age in OND. (**D**) Age-dependent shifts in monocyte subsets among pooled patients with Multiple Sclerosis (MS), showing positive correlation for CD14+CD16+ frequencies. (**C**) and (**D**): Regression lines with grey shaded bands represent 95% confidence intervals (**E**) Major immune lineage absolute counts during active versus non-active relapse, highlighting significant expansion of T cells and monocytes. (**F**) CD4+ T cell subset expansion during relapse, particularly in EM1 and EM2 populations. (**G**) CD8+ T cell changes during relapse showing increased naive and EM1 populations. (**H**) Enhanced CD38 expression on CD8+ T cells during relapse. (**A, B, E, F, G, H**) Statistical comparisons were performed using pairwise Wilcoxon rank-sum tests with Benjamini-Hochberg correction for multiple testing. Each data point represents an individual patient sample. Sample sizes: RRMS (*N* = 29), PPMS (*N* = 7), OND (*N* = 27), Relapse (*n* = 10) Non-Relapse (*n* = 19). The boxes show the interquartile range with the median line, while the whiskers extend to the largest and smallest values within 1.5 × IQR from the box edges **C**, **D**: Spearman's rank correlation.

Analysis of monocyte subsets revealed an increased frequency of CD14+CD16+ non-classical monocytes in PPMS compared with OND and RRMS [+10.4%, (IQR: ONR: 18.35–PPMS: 5.20), *P* = 0.047, +9.9%, (IQR: PPMS: 5.20—RRMS: 15.10), *P* = 0.0050] with a reciprocal pattern in CD14+CD16− monocytes (Fig. 3A). NK cell populations showed significant expansion in RRMS versus OND in both CD56+CD16+ [+18.0 cells/mL, (IQR:OND: 4.10—RRMS: 28.00), *P* < 0.0001] and CD56+CD16− subsets [+37.7 cells/mL, (IQR:OND: 13.50—RRMS: 77.00), *P* < 0.0001] (Fig. 3B).

### Relapse-associated immune cell alterations in multiple sclerosis

Comparison of RRMS patients during active relapse (*n* = 10) versus non-relapse (*n* = 19) states revealed distinct immunological profiles. During active relapse, absolute cell counts for CD3+ T cells [+4907 cells/mL, (IQR: Non-relapse: 4052.00—Relapse: 5479.75), *P* = 0.011] and CD14+ monocytes [+227 cells/mL, (IQR: Non-relapse: 261.60—Relapse: 402.02), *P* = 0.016] were higher, while their relative frequencies was unchanged (Fig. 3E).

T cell populations showed subset-specific expansions during relapse. Both CD4+ [+3371 cells/mL, (IQR: Non-relapse: 2550.00—Relapse: 3693.50), *P* = 0.016] and CD8+ T cells [+1072 cells/mL, (IQR: Non-relapse: 950.00—Relapse: 2413.75), *P* = 0.024] demonstrated increased absolute counts. Within CD4+ T cell subsets, significant elevations were observed specifically in effector memory populations: EM1 [+879 cells/mL, (IQR: Non-relapse: 991.50—Relapse: 2412.00), *P* = 0.023] and EM2 [+527 cells/mL, (IQR: Non-relapse: 392.30—Relapse: 575.60), *P* = 0.004] (Fig. 3F). The CD8+ T cell compartment showed expansion in both naive [+326 cells/mL, (IQR: Non-relapse: 147.50—Relapse: 627.28), *P* = 0.011] and EM1 [+407 cells/mL, (IQR: Non-relapse: 620.60—Relapse: 1231.95), *P* = 0.031] populations (Fig. 3G). CD38 expression on CD8+ T cells was elevated during relapse [+275 cells/mL, (IQR: Non-relapse: 208.95—Relapse: 484.12), *P* = 0.012] (Fig. 3H).

Unlike the T cell changes, B cell subsets were stable distributions between relapse states. Monocyte populations showed trends toward higher absolute numbers during relapse in both classical [+198 cells/mL, (IQR: Non-relapse: 232.45—Relapse: 343.95), *P* = 0.077] and non-classical [+38.4 cells/mL, (IQR: Non-relapse: 88.15—Relapse: 123.30), *P* = 0.094] subsets.

### Age-associated patterns in CSF immune cell populations

The characterization of MS-specific immune profiles and relapse-associated changes led us to investigate whether age might be orchestrating some of these observed immunological alterations.

Age-related changes in CSF immune cell populations exhibited distinct patterns across study groups.

In OND, absolute counts of CSF monocyte subsets showed positive correlations with age, specifically CD14+ [r_SP_ = 0.596, (95% CI: 0.279–0.796), *P* = 0.027] (Fig. 3C) and absolute CD14+CD16+ populations [r_SP_ = 0.583, (95% CI: 0.261–0.788), *P* = 0.027].

To examine age-related changes in MS, we combined groups for analysis given the age variations and the continuum of the disease.

In our pooled analysis of patients with MS, CSF immune composition showed substantial age-related changes. In absolute counts, we observed declining lymphocyte populations, with the strongest decreases in naive B cells [r_SP_ = −0.524, (95% CI: −0.727 to −0.236), *P* = 0.016], and double-negative B cells [r_SP_ = −0.517, (95% CI: −0.722 to −0.227), *P* = 0.016]. Analysis of relative frequencies revealed a shift in monocyte subsets with age, characterized by an increase in CD14+CD16+ [r_SP_ = 0.67, (95% CI: 0.437–0.818), *P* = 0.00029] and with a reciprocal pattern in CD14+CD16− monocytes. T cell composition also changed, with increased frequencies of CD8+ EM2 cells [r_SP_ = 0.62, (95% CI: 0.369–0.789), *P* < 0.0001] and TCRαβ cells [r_SP_ = 0.53, (95% CI: 0.249–0.733), *P* = 0.0060], while naive populations declined (Fig. 3D, Supplementary Figs. 2 and 3).

### Linking immune profiles to tissue damage biomarkers

After identifying immune cell alterations in patients with MS in general as well as during disease activity, we turned our focus to their potential downstream consequences on neural tissue. Specifically, we analysed two key biomarkers of tissue damage to better understand the relationship between immune dysfunction and tissue damage: NfL, indicating axonal injury, and GFAP, reflecting astrocytic activation.

**Figure 4:**
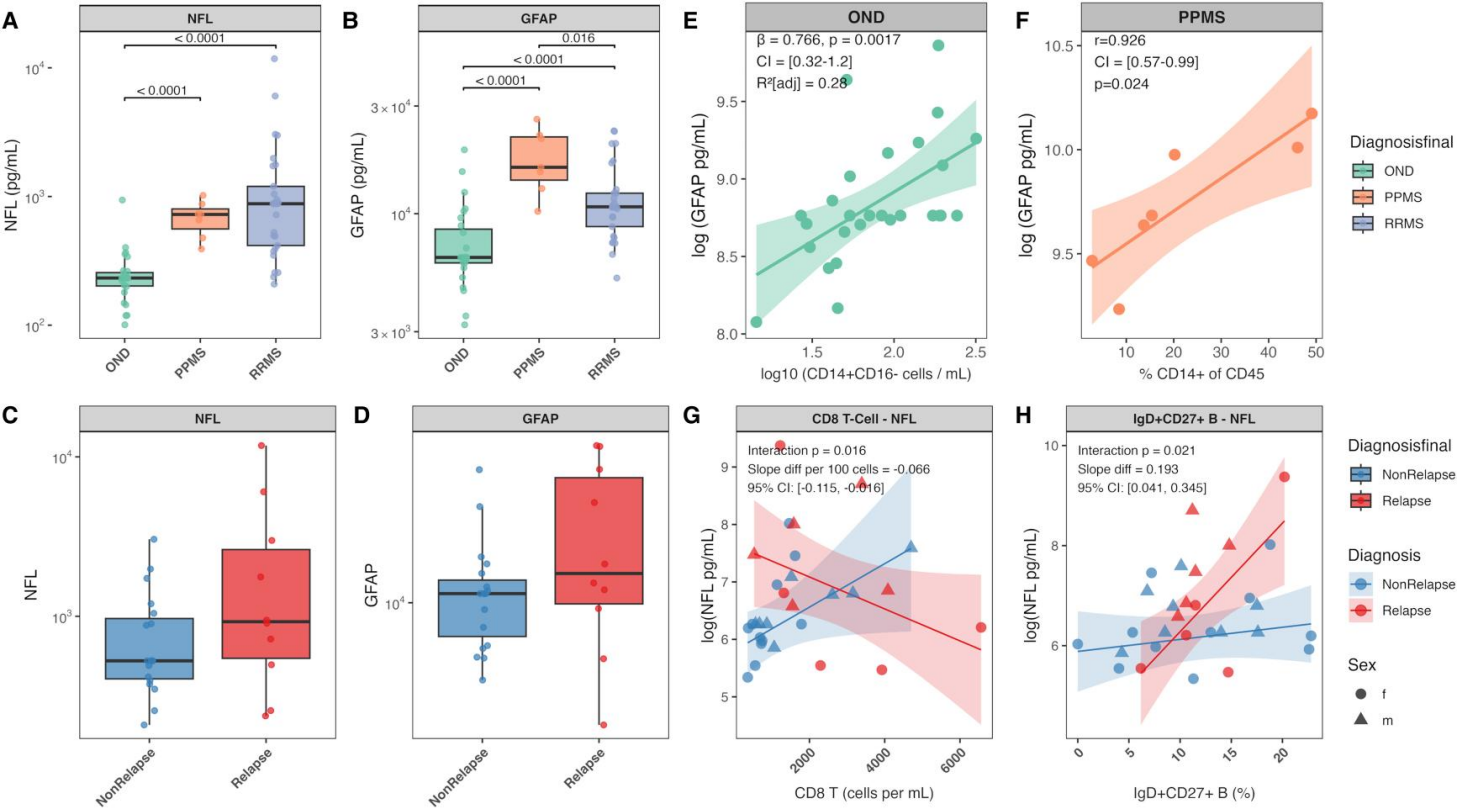
**Associations between tissue damage biomarkers and immune cell subsets across OND, MS, and disease activity.** (**A**) NfL levels across study cohorts, showing significant elevations in RRMS and PPMS patients compared with other neurological diseases (OND), with the highest levels observed in RRMS. (**B**) Glial fibrillary acidic protein (GFAP) levels across study cohorts, with PPMS patients exhibiting significantly higher levels than RRMS patients and OND. (**C**) NfL levels during relapse and non-relapse states in RRMS patients, showing a trend of elevation during relapse, though not statistically significant. (**D**) GFAP levels during relapse and non-relapse states in RRMS patients, with no significant difference observed. (**E**) Log-Log regression analysis of GFAP levels and CD14+CD16− monocyte counts in OND, adjusted for age and sex, showing a significant positive association. (**F**) Linear regression between GFAP levels and CD14+ monocyte frequencies in PPMS patients, highlighting a strong positive correlation. (**E**) and (**F**) The grey shaded band represent 95% confidence intervals. (**G**) Relationship between CD8+ T cell proportions and NfL levels, showing opposing trends during relapse (negative association) and non-relapse (positive association). (**H**) Relationship between memory B cells (CD19+IgD+CD27−) and NfL levels, with a significant stronger association observed during relapse compared with non-relapse. (**G**) and (**H**), The coloured shaded bands represent 95% confidence intervals. (**A**, **B**, **C**, **D**) Statistical comparisons were performed using pairwise Wilcoxon rank-sum tests with Benjamini-Hochberg correction for multiple testing. Each data point represents an individual patient sample. Sample sizes: RRMS (*N* = 29), PPMS (*N* = 7), OND (*N* = 27), Relapse (*n* = 10) Non-Relapse (*n* = 19). The boxes show the interquartile range with the median line, while the whiskers extend to the largest and smallest values within 1.5 × IQR from the box edges. (**E**, **F**, **G**, **H**) Multiple linear regression analyses. **G** and **H**, interaction terms were included to assess differential relationships during relapse vs. non-relapse states. Regression coefficients (β), confidence intervals, and *P*-values are reported on each plot.

Both markers showed distinct patterns across MS subtypes. NfL concentrations demonstrated significant variation across study groups, with substantial elevations in both MS subtypes compared with OND. Patients with RRMS showed the highest NfL levels [+646 pg/mL, (IQR: OND: 54.46—RRMS: 782.00), *P* < 0.0001], followed by patients with PPMS [+492 pg/mL, (IQR: OND: 54.46—PPMS: 237.06), *P* < 0.0001] (Fig. 4A). Similarly, GFAP levels varied between groups, but with a different pattern: patients with PPMS exhibited markedly higher GFAP levels [+9658 pg/mL, (IQR: OND: 2493.61—PPMS: 7756.20), *P* < 0.00031] compared with RRMS [+4325 pg/mL, (IQR OND: 2493.61—RRMS: 3561.77), *P* < 0.0001] (Fig. 4B).

In addition to subtype differences, we examined these markers during disease exacerbations in patients with RRMS. During active relapse, both markers showed moderate elevations that did not meet the set threshold for statistical significance (Fig. 4C and D).

To dissect the relationship between immune cell populations and tissue damage markers while accounting for age-related effects, we performed partial correlation and regression analyses.

In OND, we identified a robust association between classical CD14+CD16− monocytes and GFAP levels in both absolute counts [r_SP_ = 0.563, (95% CI: 0.233–0.777), *P* = 0.0082] and relative frequencies [r_SP_ = 0.66, (95% CI: 0.379–0.834), *P* < 0.0001], with a reciprocal negative correlation for CD14+CD16+ monocytes.

Log-log regression analysis, adjusted for age and sex, confirmed the relationship between CD14+CD16− monocyte counts and GFAP levels. A 10-fold increase in CD14+CD16− monocyte counts corresponded to a 2.15-fold increase in GFAP [GMR = 2.15, (95% CI: 1.377–3.364), *P* = 0.0017]. This adjusted model explained 28.4% of GFAP level variance (adjusted *R*² = 0.284, *P* = 0.013), with neither age nor sex showing significant effects (Fig. 4E).

Disease-specific patterns were observed in MS subtypes. Patients with PPMS exhibited a strong correlation between CD14+ monocyte frequency and GFAP levels [r_SP_ = 0.926, (95% CI: 0.571–0.989), *P* = 0.024] (Fig. 4F), along with multiple significant associations between T cell subsets and GFAP levels (Supplementary Table 2). In RRMS patients, NfL levels correlated positively with naive B cells count {IgD+CD27− [r_SP_ = 0.496, (95% CI: 0.159–0.730), *P* = 0.050]}. Regression analyses adjusting for age and sex revealed additional associations predominantly involving mainly B cell subsets in RRMS and T cell subsets in PPMS, nonetheless these associations did not remain significant after correction for multiple testing.

### Tissue injury markers show distinct associations with immune cell populations during relapse and non-relapse states

Given the observed patterns, we next addressed the question, whether relapses influenced the relationship between immune cell populations and neurodegeneration. In non-active relapse, NfL levels showed strong positive correlations with absolute counts of several CD8+ T cell {CD8+ T cells [r_SP_ = 0.74, (95% CI: 0.43–0.89), *P* = 0.0011], CD8+ EM, [r_SP_ = 0.74, (95% CI: 0.43–0.89), *P* = 0.0011]} and absolute counts of B cell subsets IgD+CD27− [r_SP_ = 0.71, (95% CI: 0.38–0.88), *P* = 0.067], IgD−CD27− [r_SP_ = 0.66, (95% CI: 0.29–0.68), *P* = 0.0060]. GFAP levels correlated with T cell numbers {CD3+ T [r_SP_ = 0.54, (95% CI: 0.12–0.80), *P* = 0.039]} (Supplementary Table 2). Relative proportions revealed a correlation between NfL and lineage-negative (Lin−) cells during active relapse [r_SP_ = 0.73(95% CI: 0.18–0.93), *P* = 0.026].

To investigate the differential relationship between cell subsets and NfL/GFAP levels in active versus non-active RRMS, we performed regression analyses. CD8+ T cell counts demonstrated a significant interaction with relapse status in relation to NfL levels [GMR: 0.936 (95% CI: 0.889 to 0.987), *P* = 0.016], with each 100 cells/mL increase in CD8+ T cells was associated with a 3.9% increase in NfL levels during non-relapse states [GMR: 1.039 (95% CI: 0.998 to 1.08), *P* = 0.060] and reversed during relapse (Fig. 4G). The strongest association was observed with relative proportions of naive CD4+ T cells (adjusted *R*² = 0.53, *P* = 0.0028), with notably different relationships between relapse and non-relapse states: during relapse, an increase in naive CD4+ T cell percentage was associated with a nearly three-fold increase in NfL [cumulative GMR: 2.892 (95% CI: 1.352–6.188), *P* < 0.0001], while this was reversed in non-relapse state [GMR: 0.689 (95% CI: 0.449–1.057), *P* = 0.101].

Relative proportions of CD19+ B cells exhibited an additional 55.3% increase in NfL levels during relapse compared with non-relapse states [GMR: 1.553 (95% CI: 1.076–2.241), *P* = 0.028] (Fig. 4H), while CD16+ NK cells showed reversed effects during relapse [GMR: 0.807 (95% CI: 0.716–0.910), *P* = 0.0019].

GFAP levels showed a substantial association with CD3+CD56+ cells (adjusted *R*² = 0.50, *P* = 0.0032), where during relapse, each percentage increase in CD3+CD56+ cells was associated with a 76% lower GFAP level (GMR: 0.239 [95% CI: 0.112–0.508], *P* = 0.0011) compared with non-relapse.

CD16− NK cell frequency demonstrated a similar but weaker pattern [GMR: 0.943 (95% CI: 0.904–0.984), *P* = 0.012], while Lin- cells showed an opposing relationship.

Age emerged as a significant covariate across most models, and male sex was generally associated with higher biomarker levels. Model performance varied, with adjusted *R*² values ranging from 0.32 to 0.53 for NfL models and 0.35 to 0.50 for GFAP models, suggesting differential involvement of immune cell populations in neuroinflammatory processes during relapse and remission phases.

## Discussion

Our comprehensive analysis of CSF immune cell populations in MS revealed three key findings: (i) distinct immunological signatures characterize MS disease states, with age substantially influencing CSF immune composition across the disease spectrum; (ii) classical monocytes strongly correlate with GFAP levels in OND, suggesting a previously unrecognized association that may reflect a role in CNS homeostasis; and (iii) the relationship between immune cells and tissue damage markers fundamentally shifts during MS relapses, indicating distinct pathophysiological mechanisms during different disease activity states.

Patients with RRMS showed significant lymphocyte expansion, particularly in T and B cell compartments, compared with OND and patients with PPMS. While T cell expansion aligns with previous findings, albeit from limited sample sizes, we observed an enrichment of regulatory T cells (Tregs) in RRMS patients, corroborating earlier reports.^16^ The functional significance of this Treg enrichment—including whether it is representing a compensatory anti-inflammatory mechanism—remains unclear. Recent work suggests Treg dysfunction in MS may stem from destabilized FOXP3 expression,^17^ and while these cells have been detected within MS lesions,^18^ their presence suggests intrinsic dysfunction rather than complete impairment of recruitment to sites of inflammation.

Our analysis reveals substantial age-related changes in CSF immune composition across the MS disease spectrum, supporting the concept of MS as a continuous disease process modulated by aging. In PPMS, we observed an elevation in CD14+CD16+ monocytes, consistent with previous studies.^19^ The observed elevation of CD14+CD16+ monocytes shows a strong positive correlation with age, suggesting that this reflects accelerated immunological age-related changes rather than disease-subtype specific alterations.

This elevation may reflect an increase in border-associated macrophages (BAM)/CSF macrophages as CSF transcriptomic analyses revealed elevated expression of BAM/microglial markers (including *TREM2*, *LYVE1*, and *GPR34*) in CD14+CD16+ monocytes.^20,21^ This age-related shift may represent one pathway linking aging to altered inflammatory responses in MS, possibly accounting for certain age-related changes in disease manifestation and potentially support a gradual shift to compartmentalization of inflammation.^22^

Our biomarker correlation analyses revealed notable patterns. While CD14+CD16+ populations increase with aging in OND, we identified a strong association between classical CD14+CD16− monocytes and GFAP levels in OND, accounting for 28.4% of GFAP variance. This association suggests that myeloid cells may be involved in processes related to astrocytic activation under physiological conditions, though the nature of this relationship requires further investigation.

Given the link between GFAP levels and neurodegenerative diseases,^23^ it is plausible that these cells may also play a role in disease pathology under specific conditions.

Supporting this hypothesis, recent single-cell transcriptomic analyses of aging demonstrated that CD14+CD16− monocytes undergo the most pronounced dysregulation in cognitively impaired individuals compared with OND after using reference dataset to reannotate cells.^24^

In progressive MS, GFAP levels strongly correlated with CD14+ monocyte frequency and, to a lesser extent, with T cells, consistent with potential involvement of myeloid cells in neurodegeneration, though causal relationships remain to be established and may be influenced by age-related processes. Conversely, NfL levels in RRMS associated predominantly with B cell subsets, aligning with recent data highlighting B:T cell interactions via CD27/CD70 pathway, which is disrupted by effective B cell-depleting therapies.^25^

A key finding emerged in the state-dependent relationship between immune cell populations and tissue damage markers. During non-relapse states, NfL levels strongly correlated with CD8+ T cell populations (particularly effector memory cells) and various B cell subsets, consistent with potential chronic inflammatory processes that may contribute to ongoing axonal damage even during clinical stability.^26,27^ During active relapse, these relationships shifted dramatically: CD8+ T cells showed negative NfL association, while naive CD4+ T cells demonstrated strong positive correlation, suggesting either active cell recruitment or re-expression of naïve markers by effector memory cells. Furthermore, our analysis revealed that lineage-negative cells, a heterogeneous population encompassing dendritic cell subsets and other non-conventional immune cells, showed significant associations with NfL during relapse.

Additionally, CD19+ B cells exhibited enhanced positive association with NfL levels.These immune profile shifts align with previous findings by Ghezzi *et al*.,^28^ who reported enrichment of GM-CSF and IL-13-producing CD4+ T cells in the CSF during relapse, contrasting with elevated IL-2-producing CD8+ T cells during non-relapse periods.^28^ These findings indicate a distinct shift from CD8:B cell- to CD4:B cell-driven immune mechanisms during acute exacerbations, though this could alternatively reflect cells remaining in the CSF rather than the primary inflammatory response.

From a clinical perspective, CSF immune cell profiling offers mechanistically distinct information compared with conventional tissue damage biomarkers. While NfL and GFAP measure the final downstream consequences of astrocytic activation and neurodegeneration—essentially quantifying tissue damage after it has occurred—immune cell profiling identifies the specific cellular drivers of pathological processes before irreversible damage accumulates. Our findings demonstrate that different immune cell populations correlate with tissue damage markers depending on disease state, suggesting distinct pathogenic mechanisms. This mechanistic granularity could enable personalized treatment approaches by identifying the disease-driving cells in individual patients at specific timepoints. Furthermore, the age-related immune signatures we identified, may provide early indicators of shifting pathogenic mechanisms before they manifest as measurable tissue damage in NfL or GFAP levels. This upstream cellular information could potentially guide pre-emptive therapeutic interventions, moving beyond reactive damage control toward proactive mechanism-targeted treatment.

However, clinical translation would require stepwise validation: first, demonstrating predictive value for clinical outcomes beyond current biomarkers; second, mechanistic confirmation of causal relationships between immune cells and tissue damage; and finally, prospective studies showing that immune cell-guided therapy selection improves patient outcomes.

Several limitations should be acknowledged. This study was designed as an exploratory study to identify patterns between CSF immune cells and tissue damage markers, and the sample size was not determined based on formal power calculations. While our CSF analyses provide valuable insights, they may not fully reflect the immune dynamics within the CNS parenchyma, where key pathological processes occur. Additionally, the lack of functional studies on the identified immune cell populations limits our understanding of their specific roles in disease progression. Given the exploratory nature of our study, the relatively small sample size, particularly in the PPMS group, presents two statistical concerns: it may have limited our ability to detect subtle differences, while also potentially increasing the risk of false positive findings. Therefore, our results should be interpreted with appropriate caution, and future confirmatory studies with pre-specified power calculations are needed to validate our findings.

The cross-sectional nature of our analysis prevents direct assessment of temporal relationships between immune cell changes and tissue damage progression. Further, our cohort showed heterogeneity that could impact result interpretation. Specifically, a minor fraction of patients had received methylprednisolone therapy before LP, which could affect immune cell profiles and biomarker levels. To address this potential confounding factor, we performed sensitivity analyses excluding steroid-treated patients, which showed consistent directional changes in immune cell populations (Supplementary Table 2). While our pooled analysis of MS patients helps address the age-related confound in our cohort, the uneven age distribution across traditional MS phenotypes limits our ability to disentangle disease-specific from age-related changes, despite statistical adjustments. Future studies with age-matched cohorts across the MS disease spectrum would help to better characterize how aging interacts with disease. It should be noted that our analyses were primarily adjusted for age and sex only, and additional confounding factors may have influenced the results.

In conclusion, this study provides insights into the complex relationship between immune cell dynamics and tissue damage in MS, with implications that extend beyond mere disease state classification. Our findings demonstrate that accurate interpretation of both cellular and molecular CSF biomarkers requires careful consideration of multiple factors: disease activity state alters immune cell-biomarker relationships, necessitating standardized sampling timepoints and separate analysis of relapse/non-relapse states; age substantially influences CSF immune composition, requiring careful age-matching and stratification in study design.

The discovery that immune-tissue damage correlational relationships systematically shift between disease states suggests potentially distinct pathogenic mechanisms: the predominance of CD4+ T cell and B cell associations during relapses, contrasting with CD8+ T and B cell relationships during remission, suggests separate immunological axes driving tissue damage, though functional validation is required to establish causation. If validated mechanistically, these differential correlation patterns could help explain why some MS treatments show varying efficacy across relapse and relapse-independent progression, and may support the development of distinct therapeutic strategies targeting separate immunopathogenic pathways.

The unexpected finding of strong monocyte-GFAP associations in OND identifies a previously unrecognized correlation that may indicate myeloid cell involvement in CNS homeostatic processes, though the mechanistic basis of this relationship requires further investigation.

This association between myeloid cells and tissue integrity markers suggests a potential relationship in both health and disease, though causal mechanisms remain to be determined. Future studies should investigate whether dysregulation of this homeostatic mechanism contributes to neurodegenerative processes across various CNS diseases. These insights call for mechanistic studies to delineate how different immune cell populations contribute to tissue damage and repair, potentially leading to more targeted therapeutic strategies that consider both disease state and the complex role of immune cells in CSF and CNS homeostasis.

## Supplementary Material

fcaf387_Supplementary_Data

## Data Availability

Raw anonymized data of this study are available from the corresponding author upon reasonable request by a qualified researcher and on approval by the data-clearing committees of the Medical Universities of Vienna. Statistical code and functions used in this study are provided in Supplementary Table 3. No new code or specialized, in-house scripts were generated for this study.
