# Supplementary material for: Dynamic relationship between cerebrospinal fluid immune cells and tissue damage markers in multiple sclerosis
Source: Brain Commun. 2025 Nov 5;7(6):fcaf387. doi: 10.1093/braincomms/fcaf387 (PMC12598767; doi:10.1093/braincomms/fcaf387)
Supplement: fcaf387_Supplementary_Data [file fcaf387_supplementary_data.pdf]

Supplementary Materials

Supplementary Figure 1

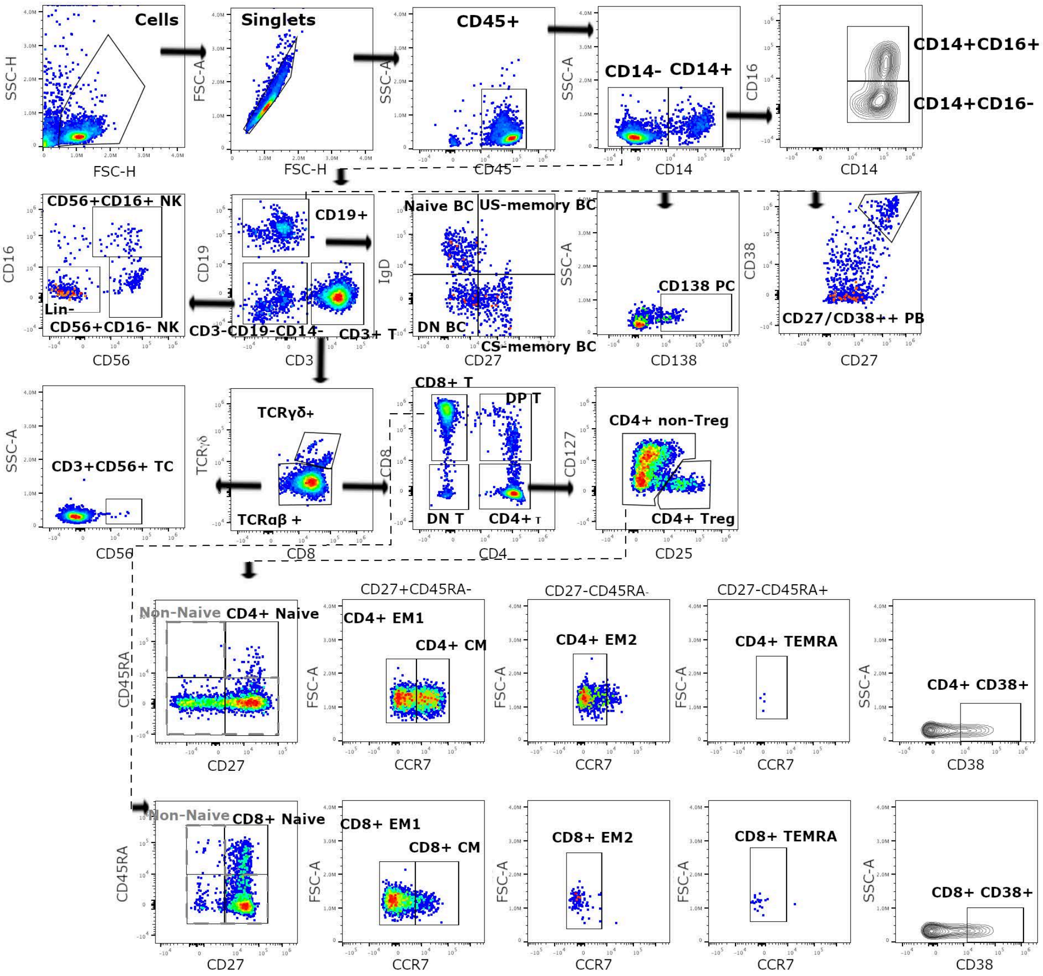

Supplementary Figure 1: Flow cytometry gating strategy for comprehensive immunophenotyping of cerebrospinal fluid (CSF) cells. The sequential gating hierarchy demonstrates the identification of distinct immune cell populations.

Supplementary Figure 2

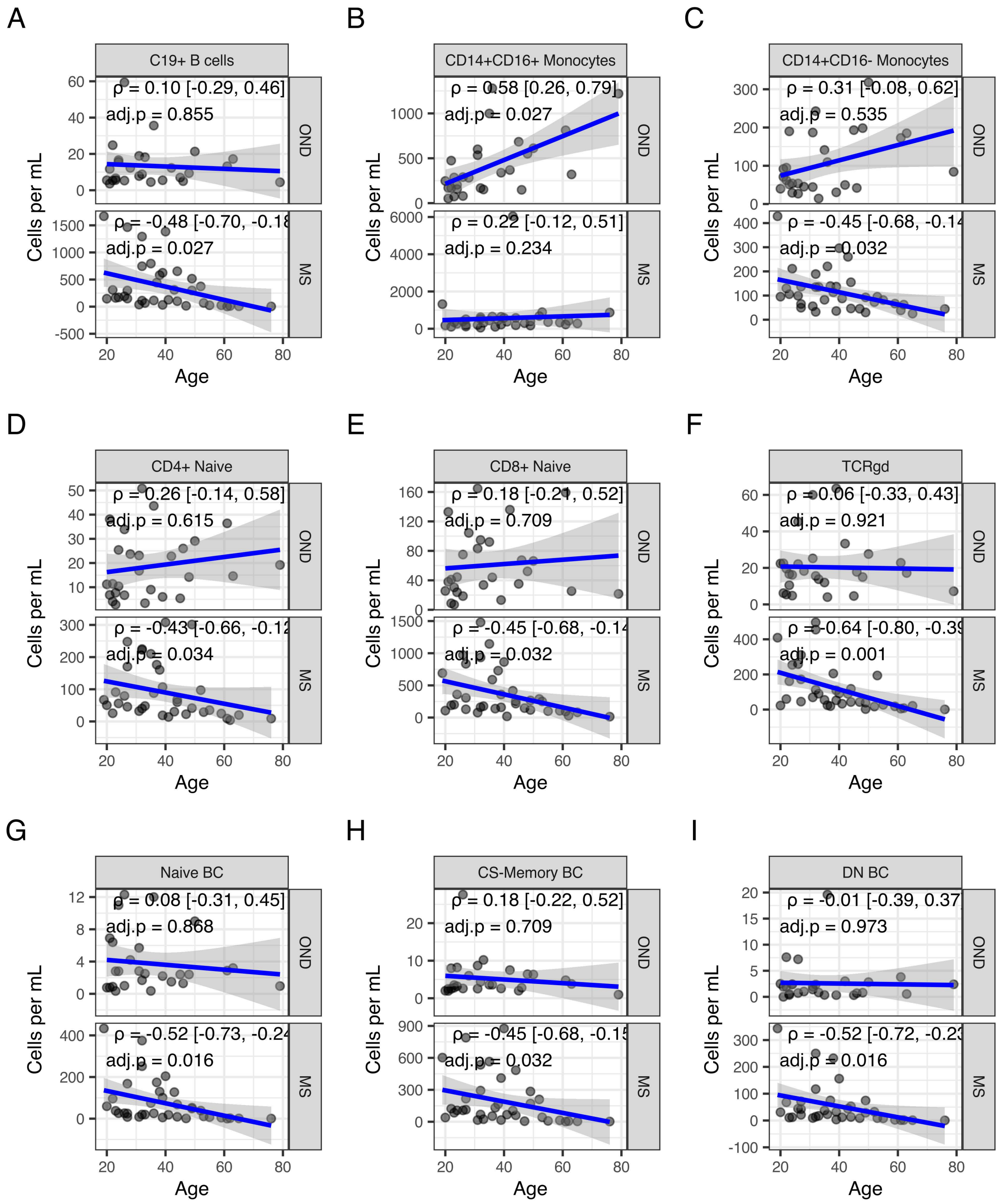

Supplementary Figure 2: Age-related changes in absolute immune cell populations across neurological diseases.
Correlation analysis between patient age and circulating immune cell subset concentrations (cells/mL) in multiple sclerosis (MS) and other neurological diseases (OND). Panels A-I show Spearman rank correlation coefficients with regression lines and 95% confidence intervals (grey shaded regions). Each point represents an individual patient sample (MS: n=36; OND: n=27). Only statistically significant correlations are displayed.

Supplementary Figure 3

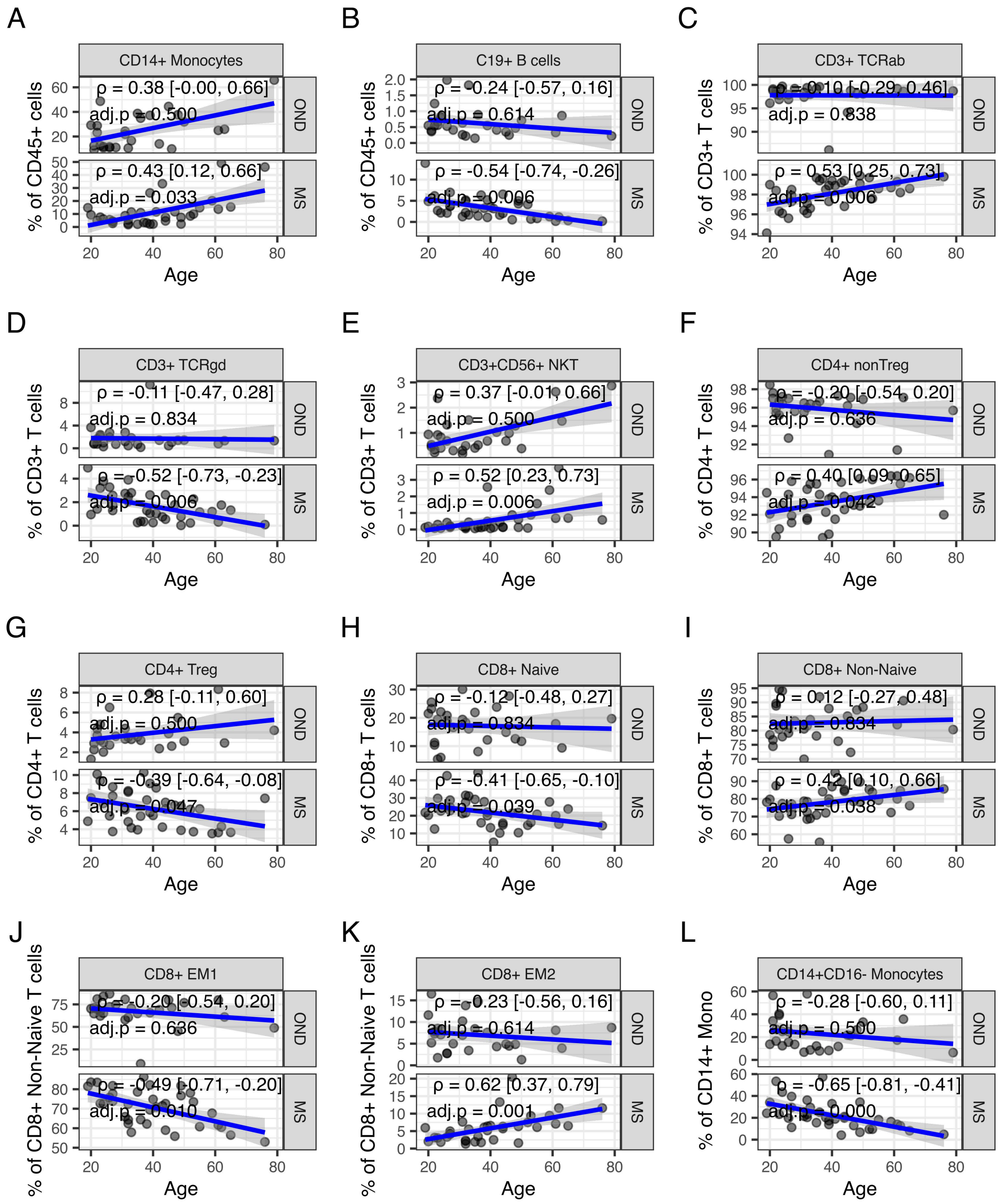

Supplementary Figure 3: Age-related changes in relative immune cell populations across neurological diseases.

Correlation analysis between patient age and relative frequencies of circulating immune cell subset (% of population) in multiple sclerosis (MS) and other neurological diseases (OND). Panels A-I show Spearman rank correlation coefficients with regression lines and 95% confidence intervals (grey shaded regions). Each point represents an individual patient sample (MS: n=36; OND: n=27). Only statistically significant correlations are displayed.

**Specificity**	**Fluorochrome**	**Catalog #**	**Vendor**
CD3	cFluor V420	R7-40000	Cytek Biosciences
CD14	cFluor V450	R7-40000	Cytek Biosciences
CD45	cFluor V547	R7-40000	Cytek Biosciences
CD138	Super Bright 600	63-1389-42	Thermo Fisher Scientific
CD8	cFluor B515	R7-40000	Cytek Biosciences
CD45RA	cFluor B690	R7-40000	Cytek Biosciences
TCRgd	PerCP-eFluor 710	46-9959-42	Thermo Fisher Scientific
CCR7	cFluor BYG575	R7-40000	Cytek Biosciences
CD38	PE-Dazzle 594	356630	BioLegend
IgD	cFluor BYG667	R7-40000	Cytek Biosciences
CD19	cFluor BYG710	R7-40000	Cytek Biosciences
CD25	cFluor BYG781	R7-40000	Cytek Biosciences
CD127	cFluor R659	R7-40000	Cytek Biosciences
CD16	cFluor R668	R7-40000	Cytek Biosciences
CD56	cFluor R720	R7-40000	Cytek Biosciences
CD4	cFluor R780	R7-40000	Cytek Biosciences
CD27	cFluor R840	R7-40000	Cytek Biosciences

Marker	Population Name	Parent Population
CD14+ Monocytes	CD14+ Monocytes	% of CD45+ Cells
CD19+ B cells	CD19+ B cells	% of CD45+ Cells
CD3+ T Cells	CD3+ T Cells	% of CD45+ Cells
CD56 NK Cells	CD56 NK Cells	% of CD45+ Cells
CD14+CD16+	CD14+CD16+ Monocytes	% of CD14+ Cells
CD14+CD16-	CD14+CD16- Monocytes	% of CD14+ Cells
CD27++CD38++	CD27/CD38++ PB	% of CD19+ Cells
IgD-CD27-	Double Negative B cells	% of CD19+ Cells
IgD-CD27+	Class-Switched Memory B cells	% of CD19+ Cells
IgD+CD27+	Non-Class-Switched Memory B cells	% of CD19+ Cells
IgD+CD27-	Naive B cells	% of CD19+ Cells
CD138+	Plasma cells	% of CD19+ Cells
TCRgd+ Cells	Gamma Delta T cells	% of CD3+ Cells
TCRgd- Cells	alpha beta T cells	% of CD3+ Cells
CD3+CD56+ T cells	CD3+CD56+ T cells	% of TCRgd- Cells
CD3+CD4+CD8- T cells	CD4 T cells	% of TCRgd- Cells
CD3+CD4-CD8+ T Cells	CD8 T cells	% of TCRgd- Cells
CD3+CD4-CD8- T cells	Double Negative T cells	% of TCRgd- Cells
CD3+CD4+CD8+ T cells	Double Positive T cells	% of TCRgd- Cells
CD4+CD127+CD25- non Treg	CD4 nonTreg T cells	% of CD4+ Cells
CD4+CD127-CD25++ Treg	CD4 Treg T cells	% of CD4+ Cells
CD4+CD45RA+CD27+ Naive	CD4 Naive T cells	% of CD4+CD127+CD25- non Treg
CD4+CD45RA-CD27+ & CD4+CD45RA-CD27- & CD4+CD45RA+CD27- Nonnaive	CD4 Non Naive T cells	% of CD4+CD127+CD25- non Treg
CD4+CD45RA-CD27+CCR7- EM1	CD4 EM1 T cells	% of CD4 Non Naive T cells
CD4+CD45RA-CD27+CCR7+ CM	CD4 CM T cells	% of CD4 Non Naive T cells
CD4+CD45RA-CD27-CCR7- EM2	CD4 EM2 T cells	% of CD4 Non Naive T cells
CD4+CD45RA+CD27-CCR7- TEMRA	CD4 TEMRA T cells	% of CD4 Non Naive T cells
CD4+CD38+ T cells	CD4+CD38+ T cells	% of CD4+ Cells
CD8+CD45RA+CD27+ Naive	CD8 Naive T cells	% of CD8 T cells
CD8+CD45RA-CD27+ & CD8+CD45RA-CD27- & CD8+CD45RA+CD27- Nonnaive	CD8 Non Naive T cells	% of CD8 T cells
CD8+CD45RA-CD27+CCR7- EM1	CD8 EM1 T cells	% of CD8 Non Naive T cells
CD8+CD45RA-CD27+CCR7+ CM	CD8 CM T cells	% of CD8 Non Naive T cells
CD8+CD45RA-CD27-CCR7- EM2	CD8 EM2 T cells	% of CD8 Non Naive T cells
CD8+CD45RA+CD27-CCR7- TEMRA	CD8 TEMRA T cells	% of CD8 Non Naive T cells
CD8+CD38+ T cells	CD8+CD38+ T cells	% of CD8+ Cells
CD56+CD16+ NK cells	CD16+ NK cells	% of CD14-CD3-CD19- Cells
CD56+CD16- NK cells	CD16- NK cells	% of CD14-CD3-CD19- Cells
CD56-CD16- Lin- cells	Lin- cells	% of CD14-CD3-CD19- Cells

Supplementary Table 1: Antibodies used for spectral flow cytometry.
Flow cytometry gating strategy for comprehensive immunophenotyping of cerebrospinal fluid (CSF) cells. Gates are applied hierarchically to identify specific cell populations based on characteristic surface marker expression, with percentages indicating the frequency of each subset within its parent population.

Supplementary Table 2A

	Partial Correlation Analysis NFL / GFAP						
	**NFL**						
cells / mL	**Group**	**Celltype**	**Definition**	**Correlation (r)**	**CI_lower**	**CI_upper**	**p value**
	RRMS	B cells	IgD+CD27-Cellperml	0.496	0.159	0.730	0.05041
	**GFAP**						
cells / mL	**Group**	**Celltype**	**Definition**	**Correlation (r)**	**CI_lower**	**CI_upper**	**p value**
	OND	Monocytes	CD14+CD16-	0.56	0.23	0.78	0.0082
	PPMS	T cells	CD3+ T	-0.93	-0.99	-0.57	0.024
	PPMS	T cells	TCRab+ T	-0.93	-0.99	-0.57	0.024
	PPMS	T cells	CD8 T	-0.88	-0.98	-0.39	0.039
	PPMS	T cells	CD8CD38+ T	-0.88	-0.98	-0.39	0.039
	PPMS	T cells	CD8 EM1 T	-0.88	-0.98	-0.39	0.039
Relative (%&)	**Group**	**Celltype**	**Definition**	**Correlation (r)**	**CI_lower**	**CI_upper**	**p value**
	OND	Monocytes	CD14+CD16+ of CD14+	-0.67	-0.83	-0.38	0.00033
	OND	Monocytes	CD14+CD16- of CD14+	0.66	0.38	0.83	0.00033
	PPMS	Lin-	Lin- o of CD14-CD3-CD19- Cells	-0.93	-0.99	-0.62	0.0061
	PPMS	Monocytes	CD14+ of CD45+	0.93	0.57	0.99	0.0241
	PPMS	T cells	CD3+ T of CD45+	-0.93	-0.99	-0.57	0.048

	log(GFAP) ~ log10(CD14+CD16-) + Age + Sex					
cells / mL		**β Coefficient**	**CI_lower**	**CI_upper**	**p value**	Group
	(Intercept)	7.595	6.86	8.36	<0.0001	
	log10(CD14+CD16-)	0.766	0.32	1.21	0.0017	
	Age	-0.003	-0.013	0.006	0.497	
	Sexm	-0.201	-0.48	0.086	0.162	
		Adjusted R2: 0.28	p value: 0.013			OND

Supplementary Table 2B

	Partial Correlation Analysis NFL / GFAP						
	**NFL**						
	**Group**	**Celltype**	**Definition**	**Correlation (r)**	**CI_lower**	**CI_upper**	**p value**
cells / mL	NonRelapse	CD8 T cell	CD8 T	0.74	0.43	0.89	0.0011
	NonRelapse	CD8 T cell	CD8+CM T	0.73	0.42	0.89	0.0011
	NonRelapse	CD8 T cell	CD8+EM T	0.74	0.43	0.89	0.0011
	NonRelapse	T cell	DP T	0.76	0.47	0.9	0.0014
	NonRelapse	B cell	IgD+CD27- B	0.71	0.38	0.88	0.0067
	NonRelapse	B cell	CD19+ B	0.66	0.3	0.86	0.007045
	NonRelapse	B cell	IgD-CD27- B	0.66	0.29	0.86	0.007045
	NonRelapse	B cell	IgD+CD27+	0.64	0.26	0.85	0.0071
	NonRelapse	B cell	IgD-CD27+ B	0.57	0.16	0.82	0.018
	NonRelapse	T cell	CD3+ T	0.59	0.19	0.82	0.0236
	NonRelapse	T cell	TCRab+ T	0.58	0.17	0.82	0.0236
	NonRelapse	CD8 T cell	CD8+CD38+ T	0.56	0.14	0.81	0.0237
	NonRelapse	T cell	DN	0.51	0.08	0.78	0.0447
Relative (%)	Relapse	Lin-	Lin- of of CD14-CD3-CD19- Cells	0.73	0.181	0.931	0.0263
	NonRelapse	T cell	CD3+CD56+ of T	-0.61	-0.835	-0.219	0.0413
	**GFAP**						
	**Group**	**Celltype**	**Definition**	**Correlation (r)**	**CI_lower**	**CI_upper**	**p value**
cells / mL	NonRelapse	T cell	CD3+ T	0.54	0.119	0.8	0.039
	NonRelapse	T cell	TCRab+ T	0.54	0.119	0.801	0.039

	Regression anaylsis				
	log(NFL) ~ CD8+ T* RelapseStatus + Age + Sex				
cells / mL		**β Coefficient**	**CI_lower**	**CI_upper**	**p value**
	(Intercept)	4.75	3.16	6.35	<0.0001
	CD8+ T (per 100 cells/mL)	0.04	-0.0016	0.08	0.059
	ActiveRelapse	1.84	0.59	3.08	0.0054
	Age	0.03	-0.01	0.07	0.114
	Sexm	0.39	-0.38	1.16	0.306
	8+ T (per 100 cells/mL):ActiveRelap	-0.07	-0.115	-0.016	0.016
		Adjusted R2: 0.2605	p value: 0.032		
	log(NFL) ~ CD8+ EM T* RelapseStatus + Age + Sex				
cells / mL		**β Coefficient**	**CI_lower**	**CI_upper**	**p value**
	(Intercept)	4.55	3.16	6.35	<0.0001
	CD8+ EM T (per 100 cells/mL)	0.08	0.00031	0.14	0.049
	ActiveRelapse	1.78	0.59	3.08	0.0051
	Age	0.03	-0.01	0.07	0.083
	Sexm	0.42	-0.38	1.16	0.265
	+ EM T(per 100 cells/mL):ActiveRel	-0.11	-0.2	-0.02	0.016
		Adjusted R2: 0.2605	p value: 0.0822		
	log(NFL) ~ CD19+%of CD45+* RelapseStatus + Age + Sex				
Relative (%)		**β Coefficient**	**CI_lower**	**CI_upper**	**p value**
	(Intercept)	4.18	2.5	5.86	<0.0001
	CD19+%of CD45+	0.1	-0.04	0.23	0.148
	ActiveRelapse	-0.59	-1.93	0.75	0.372
	Age	0.05	0.01	0.08	0.019
	Sexm	0.7	-0.00084	1.4	0.05
	CD19+%of CD45+:ActiveRelapse	0.44	0.05	0.83	0.028
		Adjusted R2: 0.35	p value: 0.0442		
	log(NFL) ~CD4+Naive of CD4+* RelapseStatus + Age + Sex				
Relative (%)		**β Coefficient**	**CI_lower**	**CI_upper**	**p value**
	(Intercept)	5.11	3.73	6.5	<0.0001
	Naive CD4 T	-0.37	-0.82	0.08	0.101
	ActiveRelapse	-1.85	-3.1	-0.59	0.006
	Age	0.04	0.01	0.07	0.0074
	Sexm	1.08	0.47	1.7	0.0013
	Naive CD4 T:ActiveRelapse	1.43	0.77	2.1	0.00017
		Adjusted R2: 0.53	p value: 0.00281		
	log(NFL) ~CD16+ NK* RelapseStatus + Age + Sex				
Relative (%)		**β Coefficient**	**CI_lower**	**CI_upper**	**p value**
	(Intercept)	4.05	2.47	5.63	<0.0001
	CD16+ NK	0.02	-0.03	0.06	0.454
	ActiveRelapse	2.4	1.14	3.67	0.001
	Age	0.05	0.01	0.09	0.0097
	Sexm	0.9	0.23	1.58	0.011
	CD16+ NK:ActiveRelapse	-0.21	-0.34	-0.09	0.0019
		Adjusted R2: 0.38	p value: 0.0175		
	log(NFL) ~Lin-* RelapseStatus + Age + Sex				
Relative (%)		**β Coefficient**	**CI_lower**	**CI_upper**	**p value**
	(Intercept)	5.08	2.45	7.71	0.001
	Lin-	-0.01	-0.03	0.02	0.569
	ActiveRelapse	-7.51	-12.64	-2.38	0.0059
	Age	0.04	0.01	0.08	0.022
	Sexm	0.78	0.09	1.48	0.029
	Lin-:ActiveRelapse	0.11	0.04	0.18	0.0034
		Adjusted R2: 0.35	p value: 0.00881		
	log(NFL) ~IgD+CD27+ of CD19 B * RelapseStatus + Age + Sex				
Relative (%)		**β Coefficient**	**CI_lower**	**CI_upper**	**p value**
	(Intercept)	5.15	3.51	6.79	<0.0001
	IgD+CD27+ of CD19 B	0.02	-0.04	0.08	0.413
	ActiveRelapse	-1.78	-3.81	0.26	0.084
	Age	0.02	-0.01	0.06	0.243
	Sexm	0.73	0.04	1.42	0.039
	gD+CD27+ of CD19 B :ActiveRelapse	0.19	0.04	0.35	0.021
		Adjusted R2: 0.32	p value: 0.0442		
	log(NFL) ~CD14+CD16- of CD14+ * RelapseStatus + Age + Sex				
Relative (%)		**β Coefficient**	**CI_lower**	**CI_upper**	**p value**
	(Intercept)	4.72	2.79	6.64	<0.0001
	CD14+CD16- of CD14+	-0.01	-0.04	0.02	0.58
	ActiveRelapse	-1.44	-2.99	0.11	0.067
	Age	0.04	0.01	0.08	0.027
	Sexm	0.99	0.26	1.72	0.01
	D14+CD16- of CD14+:ActiveRelapse	0.08	0.03	0.14	0.0063
		Adjusted R2: 0.34	p value: 0.0146		
	log(GFAP) ~CD3+CD56+ * RelapseStatus + Age + Sex				
Relative (%)		**β Coefficient**	**CI_lower**	**CI_upper**	**p value**
	(Intercept)	8.14	7.6	8.68	<0.0001
	CD3+CD56+	-0.21	-0.43	0.01	0.061
	ActiveRelapse	0.48	0.18	0.77	0.0027
	Age	0.03	0.02	0.04	0.00014
	Sexm	0.37	0.13	0.62	0.0048
	CD3+CD56+:ActiveRelapse	-1.43	-2.23	-0.64	0.0011
		Adjusted R2: 0.50	p value: 0.00325		
	log(GFAP) ~CD16- NK* RelapseStatus + Age + Sex				
Relative (%)		**β Coefficient**	**CI_lower**	**CI_upper**	**p value**
	(Intercept)	8.57	7.97	9.16	<0.0001
	CD16- NK	-0.01	-0.03	0.01	0.456
	ActiveRelapse	1.13	0.34	1.91	0.0071
	Age	0.02	0.01	0.03	0.0078
	Sexm	0.25	-0.02	0.53	0.068
	CD16- NK:ActiveRelapse	-0.06	-0.1	-0.01	0.012
		Adjusted R2: 0.35	p value: 0.0280		
	log(GFAP) ~Lin-* RelapseStatus + Age + Sex				
Relative (%)		**β Coefficient**	**CI_lower**	**CI_upper**	**p value**
	(Intercept)	7.96	6.97	8.95	<0.0001
	Lin-	0.004	-0.01	0.01	0.354
	ActiveRelapse	-2.62	-4.55	-0.7	0.0097
	Age	0.02	0.01	0.04	0.002
	Sexm	0.29	0.02	0.55	0.033
	Lin-:ActiveRelapse	0.04	0.01	0.06	0.0067
		Adjusted R2: 0.38	p value: 0.00563		

Supplementary Table 2C

Sensitivity Analysis

Non-relapse vs Relapse

Total Cohort

Celltype	Comparison	Median Difference	IQR Non-Relapse [Q1-Q3]	IQR Relapse [Q1-Q3]	p-value
CD3+ T cells	Non-relapse vs Relapse	4907	[3222 - 7274]	[7054.5 - 12534.25]	0.011
CD14+ Monocytes	Non-relapse vs Relapse	227.35	[252.1 - 513.7]	[489.975 - 892]	0.016
CD4+ Naive	Non-relapse vs Relapse	35.6	[33.6 - 99]	[51.8 - 221.25]	0.126
CD4+ Nonaive	Non-relapse vs Relapse	3276.81	[2252.995 - 4363.02]	[4668.56 - 8011.0125]	0.011
CD4+ CM	Non-relapse vs Relapse	1121.6	[813 - 2135]	[1924.5 - 4345.5]	0.056
CD4+ EM1	Non-relapse vs Relapse	879	[740 - 1731.5]	[1506 - 3918]	0.023
CD4+ EM2	Non-relapse vs Relapse	527.25	[255.2 - 647.5]	[753.5 - 1329.1]	0.004
CD4+ TEMRA	Non-relapse vs Relapse	2645	[0.42 - 6.25]	[2.445 - 6]	0.191
CD8+ Naive	Non-relapse vs Relapse	325.65	[133.7 - 281.2]	[289.6 - 916.875]	0.011
CD8+ Nonaive	Non-relapse vs Relapse	576.08	[453.38 - 1346.65]	[1023.61 - 2695.125]	0.062
CD8+ CM	Non-relapse vs Relapse	93.45	[56.3 - 230.35]	[165.55 - 505.7]	0.069
CD8+ EM1	Non-relapse vs Relapse	406.8	[360.8 - 981.4]	[808.05 - 2040]	0.031
CD8+ EM2	Non-relapse vs Relapse	16.15	[17.75 - 65]	[29.175 - 108.375]	0.302
CD8+ TEMRA	Non-relapse vs Relapse	15.68	[3.455 - 14.95]	[8.3175 - 29.025]	0.094
CD4+CD38+ T	Non-relapse vs Relapse	276.05	[223.6 - 713.7]	[505.2 - 1274.25]	0.094
CD8+CD38+ T	Non-relapse vs Relapse	275.45	[91.3 - 300.25]	[281.25 - 765.375]	0.012
CD14+CD16+ Monocyt	Non-relapse vs Relapse	198.5	[193.85 - 426.3]	[298.95 - 642.9]	0.094

Regression anaylsis

		β Coefficient	CI_lower	CI_upper	p value
log(NFL) ~ CD8+ T* RelapseStatus + Age + Sex					
cells / mL	(Intercept)	4.75	3.16	6.35	<0.0001
	CD8+ T (per 100 cells/mL)	0.04	-0.0016	0.08	0.059
	ActiveRelapse	1.84	0.59	3.08	0.0054
	Age	0.03	-0.01	0.07	0.114
	Sexm	0.39	-0.38	1.16	0.306
	CD8+ T (per 100 cells/mL):ActiveRelapse	-0.07	-0.12	-0.02	0.016
log(NFL) ~ CD8+ EM T* RelapseStatus + Age + Sex		β Coefficient	CI_lower	CI_upper	p value
cells / mL	(Intercept)	4.55	3.16	6.35	<0.0001
	CD8+ EM T (per 100 cells/mL)	0.08	0.00031	0.14	0.049
	ActiveRelapse	1.78	0.59	3.08	0.0051
	Age	0.03	-0.01	0.07	0.083
	Sexm	0.42	-0.38	1.16	0.265
	CD8+ EM T(per 100 cells/mL):ActiveRelapse	-0.11	-0.2	-0.02	0.016
log(NFL) ~ CD19+%of CD45+* RelapseStatus + Age + Sex		β Coefficient	CI_lower	CI_upper	p value
Relative (%)	(Intercept)	4.18	2.5	5.86	<0.0001
	CD19+%of CD45+	0.1	-0.04	0.23	0.148
	ActiveRelapse	-0.59	-1.93	0.75	0.372
	Age	0.05	0.01	0.08	0.019
	Sexm	0.7	-0.00084	1.4	0.05
	CD19+%of CD45+:ActiveRelapse	0.44	0.05	0.83	0.028
log(NFL) ~CD4+Naive of CD4+* RelapseStatus + Age + Sex		β Coefficient	CI_lower	CI_upper	p value
Relative (%)	(Intercept)	5.11	3.73	6.5	<0.0001
	Naive CD4 T	-0.37	-0.82	0.08	0.101
	ActiveRelapse	-1.85	-3.1	-0.59	0.006
	Age	0.04	0.01	0.07	0.0074
	Sexm	1.08	0.47	1.7	0.0013
	Naive CD4 T:ActiveRelapse	1.43	0.77	2.1	0.00017
log(NFL) ~CD16+ NK* RelapseStatus + Age + Sex		β Coefficient	CI_lower	CI_upper	p value
Relative (%)	(Intercept)	4.05	2.47	5.63	<0.0001
	CD16+ NK	0.02	-0.03	0.06	0.454
	ActiveRelapse	2.4	1.14	3.67	0.001
	Age	0.05	0.01	0.09	0.0097
	Sexm	0.9	0.23	1.58	0.011
	CD16+ NK:ActiveRelapse	-0.21	-0.34	-0.09	0.0019
log(NFL) ~Lin-* RelapseStatus + Age + Sex		β Coefficient	CI_lower	CI_upper	p value
Relative (%)	(Intercept)	5.08	2.45	7.71	0.001
	Lin-	-0.01	-0.03	0.02	0.569
	ActiveRelapse	-7.51	-12.64	-2.38	0.0059
	Age	0.04	0.01	0.08	0.022
	Sexm	0.78	0.09	1.48	0.029
	Lin-:ActiveRelapse	0.11	0.04	0.18	0.0034
log(NFL) ~IgD+CD27+ of CD19 B * RelapseStatus + Age + Sex		β Coefficient	CI_lower	CI_upper	p value
Relative (%)	(Intercept)	5.15	3.51	6.79	<0.0001
	IgD+CD27+ of CD19 B	0.02	-0.04	0.08	0.413
	ActiveRelapse	-1.78	-3.81	0.26	0.084
	Age	0.02	-0.01	0.06	0.243
	Sexm	0.73	0.04	1.42	0.039
	IgD+CD27+ of CD19 B :ActiveRelapse	0.19	0.04	0.35	0.021
log(NFL) ~CD14+CD16- of CD14+ * RelapseStatus + Age + Sex		β Coefficient	CI_lower	CI_upper	p value
Relative (%)	(Intercept)	4.72	2.79	6.64	<0.0001
	CD14+CD16- of CD14+	-0.01	-0.04	0.02	0.58
	ActiveRelapse	-1.44	-2.99	0.11	0.067
	Age	0.04	0.01	0.08	0.027
	Sexm	0.99	0.26	1.72	0.01
	CD14+CD16- of CD14+:ActiveRelapse	0.08	0.03	0.14	0.0063
log(GFAP) ~CD3+CD56+ * RelapseStatus + Age + Sex		β Coefficient	CI_lower	CI_upper	p value
Relative (%)	(Intercept)	8.14	7.6	8.68	<0.0001
	CD3+CD56+	-0.21	-0.43	0.01	0.061
	ActiveRelapse	0.48	0.18	0.77	0.0027
	Age	0.03	0.02	0.04	0.00014
	Sexm	0.37	0.13	0.62	0.0048
	CD3+CD56+:ActiveRelapse	-1.43	-2.23	-0.64	0.0011
log(GFAP) ~CD16- NK* RelapseStatus + Age + Sex		β Coefficient	CI_lower	CI_upper	p value
Relative (%)	(Intercept)	8.57	7.97	9.16	<0.0001
	CD16- NK	-0.01	-0.03	0.01	0.456
	ActiveRelapse	1.13	0.34	1.91	0.0071
	Age	0.02	0.01	0.03	0.0078
	Sexm	0.25	-0.02	0.53	0.068
	CD16- NK:ActiveRelapse	-0.06	-0.1	-0.01	0.012
log(GFAP) ~Lin-* RelapseStatus + Age + Sex		β Coefficient	CI_lower	CI_upper	p value
Relative (%)	(Intercept)	7.96	6.97	8.95	<0.0001
	Lin-	0.004	-0.01	0.01	0.354
	ActiveRelapse	-2.62	-4.55	-0.7	0.0097
	Age	0.02	0.01	0.04	0.002
	Sexm	0.29	0.02	0.55	0.033
	Lin-:ActiveRelapse	0.04	0.01	0.06	0.0067

Excluding patients receiving HDMP treatment prior LP

Celltype	Comparison	Median Difference	IQR Non-Relapse [Q1-Q3]	IQR Relapse [Q1-Q3]	p-value
CD3+ T cells	Non-relapse vs Relapse	5869	[3222 - 7274]	[8203 - 17280]	0.025
CD14+ Monocytes	Non-relapse vs Relapse	86.6	[252.1 - 513.7]	[391.8 - 853.75]	0.094
CD4+ Naive	Non-relapse vs Relapse	162	[33.6 - 99]	[64.8 - 263.1]	0.041
CD4+ Nonaive	Non-relapse vs Relapse	3874.35	[2252.995 - 4363.02]	[5468.96 - 12249.955]	0.025
CD4+ CM	Non-relapse vs Relapse	1077.6	[813 - 2135]	[1949 - 4872.5]	0.107
CD4+ EM1	Non-relapse vs Relapse	1344	[740 - 1731.5]	[1641 - 7255.5]	0.028
CD4+ EM2	Non-relapse vs Relapse	460.5	[255.2 - 647.5]	[681.9 - 1526]	0.035
CD4+ TEMRA	Non-relapse vs Relapse	2.73	[0.42 - 6.25]	[2.965 - 10.8]	0.224
CD8+ Naive	Non-relapse vs Relapse	687.2	[133.7 - 281.2]	[258.7 - 953.25]	0.041
CD8+ Nonaive	Non-relapse vs Relapse	582.08	[453.38 - 1346.65]	[1129.14 - 2615.25]	0.107
CD8+ CM	Non-relapse vs Relapse	82.9	[56.3 - 230.35]	[148.7 - 605]	0.12
CD8+ EM1	Non-relapse vs Relapse	428.8	[360.8 - 981.4]	[851.7 - 1935]	0.055
CD8+ EM2	Non-relapse vs Relapse	18.7	[17.75 - 65]	[40.8 - 88.75]	0.355
CD8+ TEMRA	Non-relapse vs Relapse	14.98	[3.455 - 14.95]	[9.385 - 28.05]	0.188
CD4+CD38+ T	Non-relapse vs Relapse	923	[223.6 - 713.7]	[620.25 - 1912.5]	0.015
CD8+CD38+ T	Non-relapse vs Relapse	546.9	[91.3 - 300.25]	[285.5 - 860]	0.021
CD14+CD16+ Monocytes	Non-relapse vs Relapse	129	[193.85 - 426.3]	[287.25 - 601]	0.254

Regression anaylsis

		β Coefficient	CI_lower	CI_upper	p value
log(NFL) ~ CD8+ T* RelapseStatus + Age + Sex					
cells / mL	(Intercept)	4.63	2.88	6.39	<0.0001
	CD8+ T (per 100 cells/mL)	0.04	-0.0024	0.08	0.06
	ActiveRelapse	1.82	0.34	3.29	0.018
	Age	0.03	-0.01	0.07	0.11
	Sexm	0.34	-0.56	1.23	0.44
	CD8+ T (per 100 cells/mL):ActiveRelapse	-0.07	-0.12	-0.01	0.03
log(NFL) ~ CD8+ EM T* RelapseStatus + Age + Sex		β Coefficient	CI_lower	CI_upper	p value
cells / mL	(Intercept)	4.41	2.63	6.19	<0.0001
	CD8+ EM T (per 100 cells/mL)	0.08	0	0.16	0.049
	ActiveRelapse	1.79	0.39	3.19	0.015
	Age	0.04	-0.004	0.08	0.077
	Sexm	0.35	-0.54	1.23	0.42
	CD8+ EM T(per 100 cells/mL):ActiveRelapse	-0.12	-0.22	-0.02	0.025
log(NFL) ~ CD19+%of CD45+* RelapseStatus + Age + Sex		β Coefficient	CI_lower	CI_upper	p value
Relative (%)	(Intercept)	3,51	1,93	5,09	<0.0001
	CD19+%of CD45+	0,12	0	0,24	0,056
	ActiveRelapse	-1,69	-3,21	-0,17	0,031
	Age	0,06	0,026	0,1	0,0016
	Sexm	0,74	0,072	1,4	0,03
	CD19+%of CD45+:ActiveRelapse	0,68	0,28	1,08	0,0022
log(NFL) ~CD4+Naive of CD4+* RelapseStatus + Age + Sex		β Coefficient	CI_lower	CI_upper	p value
Relative (%)	(Intercept)	4.89	3.57	6.2	<0.0001
	Naive CD4 T	-0.34	-0.75	0.08	0.11
	ActiveRelapse	-2.44	-3.73	-1.15	0.00081
	Age	0.05	0.02	0.08	0.0021
	Sexm	0.98	0.38	1.59	0.0029
	Naive CD4 T:ActiveRelapse	1.61	0.97	2.25	<0.0001
log(NFL) ~CD16+ NK* RelapseStatus + Age + Sex		β Coefficient	CI_lower	CI_upper	p value
Relative (%)	(Intercept)	3.82	2.13	5.51	<0.0001
	CD16+ NK	0.02	-0.03	0.06	0.49
	ActiveRelapse	2.5	1.07	3.93	0.0016
	Age	0.06	0.02	0.1	0.0073
	Sexm	0.96	0.21	1.7	0.015
	CD16+ NK:ActiveRelapse	-0.22	-0.35	-0.08	0.0031
log(NFL) ~Lin-* RelapseStatus + Age + Sex		β Coefficient	CI_lower	CI_upper	p value
Relative (%)	(Intercept)	4.18	1.59	6.76	0.003
	Lin-	-0.005	-0.03	0.02	0.69
	ActiveRelapse	-8.9	-13.86	-3.95	0.0013
	Age	0.06	0.02	0.1	0.0028
	Sexm	1	0.3	1.71	0.0078
	Lin-:ActiveRelapse	0.13	0.06	0.19	0.0007
log(NFL) ~IgD+CD27+ of CD19 B * RelapseStatus + Age + Sex		β Coefficient	CI_lower	CI_upper	p value
Relative (%)	(Intercept)	4.96	3.11	6.82	<0.0001
	IgD+CD27+ of CD19 B	0.02	-0.04	0.09	0.43
	ActiveRelapse	-1.63	-3.92	0.66	0.15
	Age	0.02	-0.02	0.07	0.21
	Sexm	0.8	0.005	1.59	0.0488
	IgD+CD27+ of CD19 B :ActiveRelapse	0.18	0.0001	0.36	0.0499
log(NFL) ~CD14+CD16- of CD14+ * RelapseStatus + Age + Sex		β Coefficient	CI_lower	CI_upper	p value
Relative (%)	(Intercept)	4.75	2.74	6.77	<0.0001
	CD14+CD16- of CD14+	-0.01	-0.05	0.03	0.55
	ActiveRelapse	-2.19	-4.3	-0.08	0.043
	Age	0.04	0.003	0.08	0.038
	Sexm	1.04	0.22	1.86	0.016
	CD14+CD16- of CD14+:ActiveRelapse	0.11	0.03	0.19	0.0086
log(GFAP) ~CD3+CD56+ * RelapseStatus + Age + Sex		β Coefficient	CI_lower	CI_upper	p value
Relative (%)	(Intercept)	8.19	7.74	8.64	<0.0001
	CD3+CD56+	-0.2	-0.38	-0.01	0.036
	ActiveRelapse	0.37	0.1	0.64	0.01
	Age	0.03	0.02	0.04	<0.0001
	Sexm	0.38	0.16	0.6	0.0016
	CD3+CD56+:ActiveRelapse	-1.48	-2.16	-0.79	0.00021
log(GFAP) ~CD16- NK* RelapseStatus + Age + Sex		β Coefficient	CI_lower	CI_upper	p value
Relative (%)	(Intercept)	8.36	7.83	8.89	<0.0001
	CD16- NK	-0.01	-0.03	0.01	0.21
	ActiveRelapse	1.25	0.51	2	0.0022
	Age	0.03	0.01	0.04	0.00049
	Sexm	0.38	0.13	0.63	0.0046
	CD16- NK:ActiveRelapse	-0.07	-0.11	-0.03	0.0015
log(GFAP) ~Lin-* RelapseStatus + Age + Sex		β Coefficient	CI_lower	CI_upper	p value
Relative (%)	(Intercept)	7.85	6.89	8.8	<0.0001
	Lin-	0.004	-0.01	0.01	0.27
	ActiveRelapse	-2.57	-4.41	-0.74	0.0083
	Age	0.03	0.01	0.04	0.0013
	Sexm	0.35	0.08	0.61	0.0121
	Lin-:ActiveRelapse	0.03	0.01	0.06	0.0079

Supplementary Table 2D

Cells per mL

Group	*Celltype*	*Median*	*Q1*	*Q3*	*IQR*
RRMS	CD3+ T cells	6688	3488	10308	6820
RRMS	CD4+ T	4688	2695	7372	4677
RRMS	CD4+ Treg	274,5	168	456	288
RRMS	CD8+ T	1320	690	2295	1605
RRMS	Lin- Cells	315	171,9	609	437,1
RRMS	TCRgd	89,3	42,9	172	129,1
RRMS	C19+ B cells	189,9	110,5	581,4	470,9
RRMS	CD14+ Mono	460	270	737	467
PPMS	CD3+ T cells	1614	1258	4563	3305
PPMS	CD4+ T	1110	967	3172	2205
PPMS	CD4+ Treg	41	38,7	194,05	155,35
PPMS	CD56+ NK ce	18,2	13,76	45,7	31,94
PPMS	CD8+ T	380	266,5	1218,5	952
PPMS	Lin- Cells	61,4	49,8	195,5	145,7
PPMS	TCRgd	17,1	6,6	19,8	13,2
PPMS	C19+ B cells	11,6	5,2	199,45	194,25
PPMS	CD14+ Mono	491	356	672,45	316,45
OND	CD3+ T cells	1626	797	2245,5	1448,5
OND	CD4+ T	1196	601	1627,5	1026,5
OND	CD4+ Treg	33,6	20,4	46,65	26,25
OND	CD56+ NK ce	18,8	10,75	28,45	17,7
OND	CD8+ T	360	153	530	377
OND	Lin- Cells	110,4	71,35	130,2	58,85
OND	TCRgd	17,2	11,25	22,65	11,4
OND	C19+ B cells	9,4	5,5	16,9	11,4
OND	CD14+ Mono	420	234	741,5	507,5

% of CD45+ Cells

Group	*Celltype*	*Median*	*Q1*	*Q3*	*IQR*
RRMS	CD3+ T cells	84,8	80,6	87,2	6,6
RRMS	CD14+ Mono	6,07	3,46	11	7,54
RRMS	C19+ B cells	3,18	1,66	4,97	3,31
RRMS	CD56+ NK ce	1,12	0,73	1,74	1,01
PPMS	CD3+ T cells	80,7	62,9	82,9	20
PPMS	CD14+ Mono	15,4	11,09	33,15	22,06
PPMS	C19+ B cells	1,16	0,26	2,71	2,45
PPMS	CD56+ NK ce	0,91	0,725	1,175	0,45
OND	CD3+ T cells	70,8	59,55	80,1	20,55
OND	CD14+ Mono	23,2	11,7	33,4	21,7
OND	CD56+ NK ce	0,94	0,66	1,41	0,75

Supplementary Table 2E

Age Correlation Analysis: Cells per mL

	Group	*Celltype*	*rho*	*CI lower*	*CI upper*	*padj*
Cells per mL	OND	CD14+CD16+ Monocytes	0,583	0,260	0,788	0,027
	MS	Naive BC	-0,524	-0,727	-0,236	0,016
	MS	CS-Memory BC	-0,453	-0,680	-0,146	0,032
	MS	DN BC	-0,517	-0,723	-0,227	0,016
	MS	CD4+ Naive	-0,429	-0,664	-0,117	0,034
	MS	CD8+ Naive	-0,452	-0,679	-0,145	0,032
	MS	C19+ B cells	-0,483	-0,700	-0,183	0,027
	MS	CD14+CD16- Monocytes	-0,449	-0,678	-0,141	0,032
	MS	TCRgd	-0,639	-0,800	-0,394	0,001

Age Correlation Analysis: Relative Frequency

	Group	*Celltype*	*rho*	*CI lower*	*CI upper*	*padj*
Relative (%)	MS	CD3+ TCRab	0,534	0,249	0,734	0,006
	MS	CD3+ TCRgd	-0,522	-0,726	-0,233	0,006
	MS	CD3+CD56+ NKT	0,520	0,232	0,725	0,006
	MS	CD4+ nonTreg	0,405	0,088	0,647	0,042
	MS	CD4+ Treg	-0,394	-0,640	-0,075	0,047
	MS	CD14+ Monocytes	0,430	0,119	0,665	0,033
	MS	C19+ B cells	-0,540	-0,738	-0,257	0,006
	MS	CD8+ Naive	-0,414	-0,653	-0,098	0,039
	MS	CD8+ Non-Naive	0,419	0,105	0,657	0,038
	MS	CD8+ EM1	-0,494	-0,708	-0,198	0,010
	MS	CD8+ EM2	0,623	0,370	0,790	0,001
	MS	CD14+CD16- Monocytes	-0,654	-0,809	-0,414	0,000
	MS	CD14+CD16+ Monocytes	0,670	0,438	0,818	0,000

Supplemenatary Table 3

R funtions used

packages: gtsummary V.2.0.4; rstatix V.0.7.2; ppcor V.1.1; car V.3.1.3; dplyr V.1.1.4; tidyverse V.2.0.0; ggsignif

```
tbl_summary(data, by = X) %>%  add_overall() %>%  add_p(test = list(all_continuous() ~
"kruskal.test", all_categorical() ~ "fisher.test"))
kruskal.test(celltype ~ diagnosis, data = dataset)
dunn_test(dataset$celltype, dataset$diagnosis, method = "holm")
pairwise_wilcox_test(dataset, celltype ~ diagnosis, p.adjust.method = "BH")
fisher.test(table(dataset$X, dataset$diagnosis))
cor.test(dataset$age, dataset$celltype, method = "spearman")
pcor.test(dataset$biomarker, dataset$celltype, dataset$age, method = "spearman")
shapiro.test(dataset$biomarker)
hist(dataset$biomarke)
dataset$log_biomarker <- log(dataset$biomarker)
dataset$log10_celltype <- log10(dataset$celltype)
model <- lm(log_biomarker ~ celltype + age + sex, data = dataset)
summary(model)
model <- lm(log_biomarker ~ log10_celltype + age + sex, data = dataset)
lm(log_biomarker ~ celltype * status + age + sex, data = dataset)
avPlots(model)
qqPlot(model)
coef_results <- summary(model)$coefficients
conf_intervals <- confint(model)
geometric_mean_ratios <- exp(coef(model))
geometric_ci <- exp(confint(model))
adjusted_r_squared <- summary(model)$adj.r.squared
p_values <- c(summary(model)$coefficients[2, "Pr(>|t|)"], .... )
adjusted_p_values <- p.adjust(p_values, method = "BH")
```
